# Supplementary material for: The 8-17 DNAzyme can operate in a single active structure regardless of metal ion cofactor
Source: Nat Commun. 2024 May 17;15:4218. doi: 10.1038/s41467-024-48638-x (PMC11101458; doi:10.1038/s41467-024-48638-x)
Supplement: Supplementary file 1 — Supplementary Information [file 41467_2024_48638_MOESM1_ESM.pdf]

Supplementary information

The 8-17 DNAzyme can operate in a single active structure regardless of metal ion cofactor

Julia Wieruszewska,[1,†] Aleksandra Pawłowicz,[1,†] Ewa Połomska,[1] Karol Pasternak,[1] Zofia Gdaniec,[1] Witold Andrałojć[1,*]

[1]Institute of Bioorganic Chemistry, Polish Academy of Sciences, 61-704 Poznan, Noskowskiego 12/14, Poland

* To whom correspondence should be addressed. Tel: (+4861) 8528503; Email: wandralojc@ibch.poznan.pl

[†]These authors contributed equally to this work

Table of contents:

- Supplementary Figure1. 8-17 DNAzyme construct optimization 3

- Supplementary Figure2. Catalytic reactions in presence of different M^{2+} cofactors 4

- Supplementary Figure 3. Observed rate constant k_{obs} dependence on M^{2+} concentrations 5

- Supplementary Figure 4. 8-17 short folding by Zn^{2+}at pH 7 6

- Supplementary Figure 5. Resonance assignment through isotopic labeling 7

- Supplementary Figure 6. Long-range NOEs 8

- Supplementary Figure 7. NMR spectra of 8-17_short with 4 mM Zn^{2+} and 200 mM NaCl 9

- Supplementary Figure 8. NMR spectra of 8-17_short with single types of metal ions 12

- Supplementary Figure 9. UV spectra for both 8-17 constructs with Zn^{2+}, Na^{+}, Mg^{2+}, and Pb^{2+} 15

- Supplementary Figure 10. CD and UV spectra of TBA titration with Pb^{2+} 16

- Supplementary Figure 11. CD and UV spectra a 8-17 construct with $Pb^{2+}$and Zn^{2+} 17

- Supplementary Figure 12. 1D NMR-monitored titrations of the “crystallographic” 8-17 DNAzyme 18

- Supplementary Figure 13. Catalytic activity in presence of both Pb^{2+} and Mg^{2+} 19

- Supplementary Figure 14. CSPs for 8-17_short upon Zn^{2+} titration 20

- Supplementary Figure 15. CD titrations of mutated variants of 8-17_short with Zn^{2+} 21

- Supplementary Table 1. Structure determination statistics for 8-17 DNAzyme 22

- Supplementary Table 2. Long-range NOE of the catalytic domain 23

- Supplementary Note 1. 8-17 DNAzyme construct optimization for NMR studies 25

- Supplementary Note 2. NMR data for the 8-17 short in the structure determination conditions 25

- Supplementary Note 3. NMR observations for samples containing Na^{+}, Mg^{2+}, Zn^{2+} or Pb^{2+} 27

- Supplementary Note 4. Analysis of CD-titrations of phosphorothioate (PS) modified constructs 29

- Supplementary Discussion 1. Second structural transition at higher Pb^{2+}-to-DNA molar ratios 29

- Supplementary Discussion 2. Previous FRET and CD spectroscopy results revisited 30

- Supplementary References 32

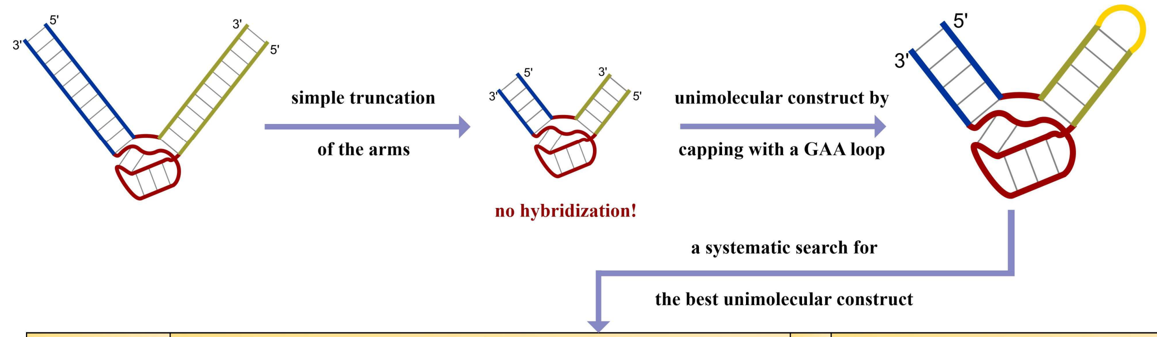

Construct name	Sequence*	nt	Result
4-4a	5’ - CCGT GG CTGC GAA GCAG TGCCAGCGGCTCGA ACGG - 3'	35	single form, sharp up to 20 °C
4-4b	5’ - CCCG GG CTGC GAA GCAG TGCCAGCGGCTCGA CGGG - 3'	35	single form, very thermally unstable
5-4	5’ - GCCCG GG CTGC GAA GCAG TGCCAGCGGCTCGA CGGGC - 3'	37	single form, improved stability, too long
6-2 GC	5’ - CCGCCG GG GC GAA GC TGCCAGCGGCTCGA CGGCGG - 3'	35	single form, sharp up to 30 °C
6-2 GG	5’ - CGCCCG GG GG GAA CC TGCCAGCGGCTCGA CGGGCG - 3'	35	multiple forms
5-3 CCC	5’ - CCCGC GG CCC GAA GGG TGCCAGCGGCTCGA GCGGG - 3'	35	single form, sharp up to 35 °C
5-3 CTG	5’ - CGCCG GG CTG GAA CAG TGCCAGCGGCTCGA CGGCG - 3'	35	multiple forms
5-3 GTC	5’ - CGCCG GG GTC GAA GAC TGCCAGCGGCTCGA CGGCG - 3'	35	single form, sharp up to 35 °C

*color coded according to secondary structure elements: blue and green (arms), red (catalytic domain), yellow (GAA loop)

Supplementary Figure 1. The general scheme of 8-17 DNAzyme construct optimization for NMR and a table of constructs tested experimentally.

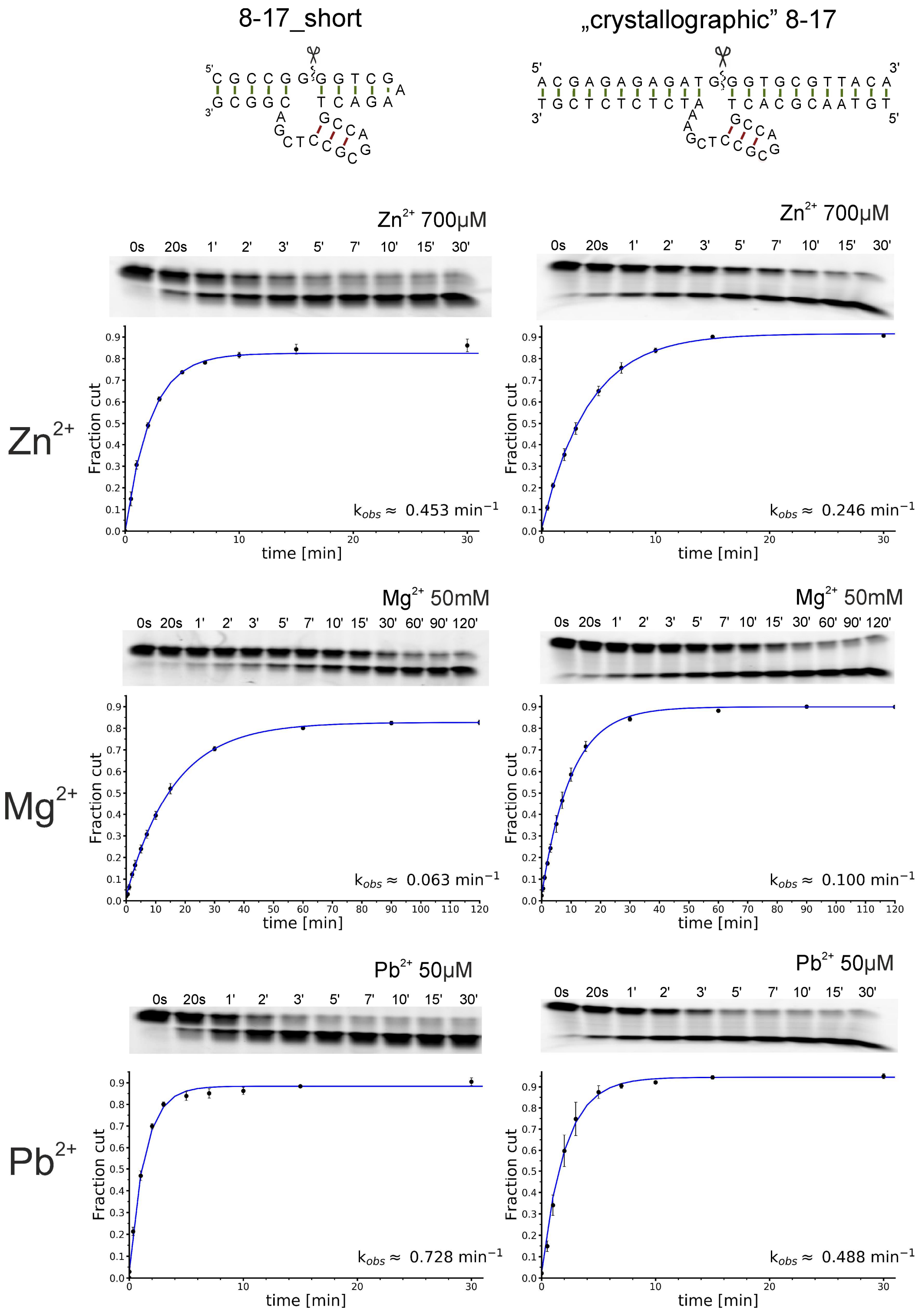

Supplementary Figure 2. The determination of RNA cleavage rate constants k_{obs} for 8-17_short (left panels) and full-length bimolecular construct of 8-17 DNAzyme (right panels) in the presence of Zn^{2+}, Mg^{2+} and Pb^{2+} monitored by gel electrophoresis. An example gel image obtained in each given conditions is given above the graphs. DNAzyme concentration of 40 µM was used throughout the measurements. The error bars represent standard deviations observed over n=3 independent experiments. Source data are provided as a Source Data file.

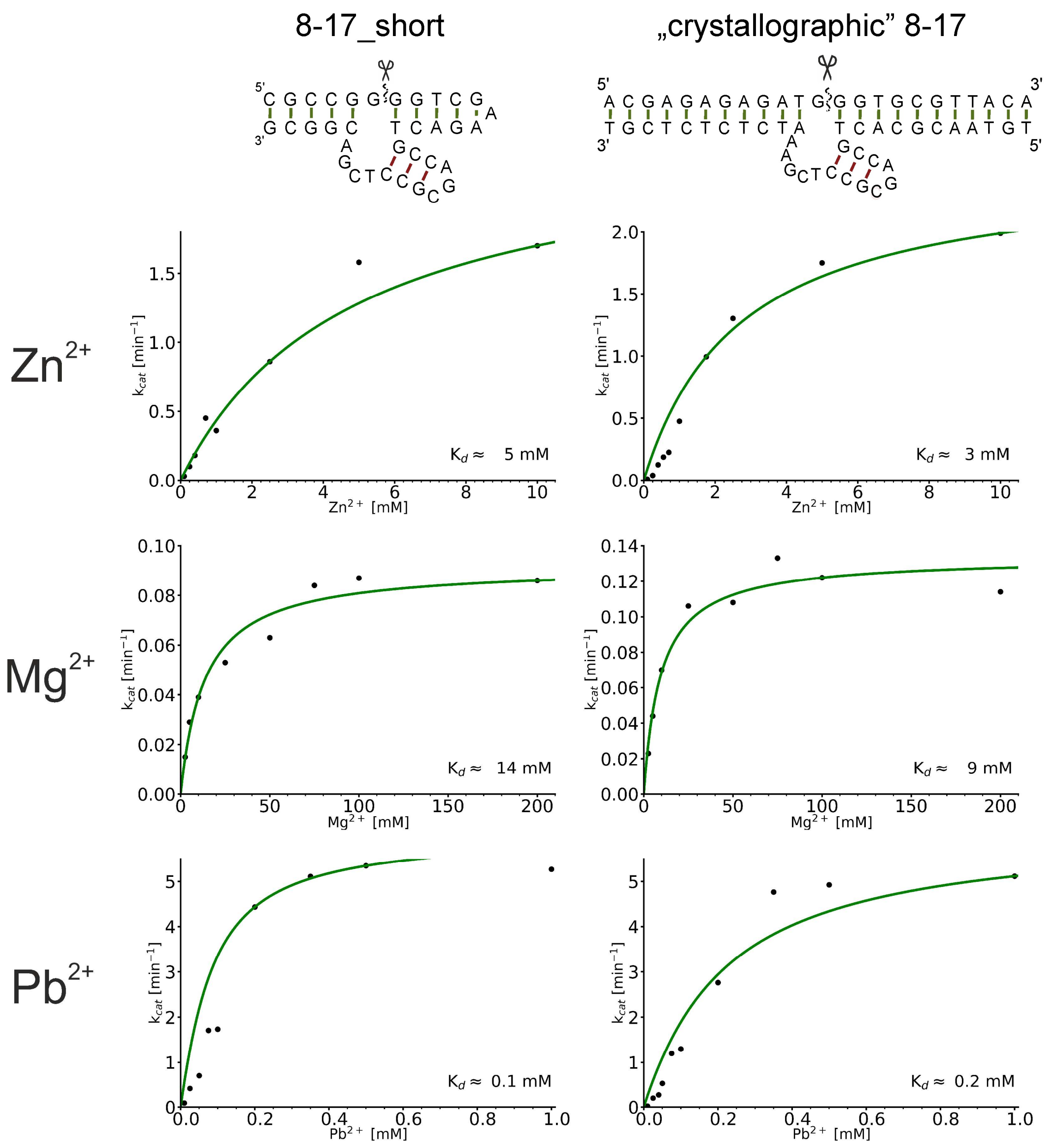

Supplementary Figure 3 The RNA cleavage rates (k_{obs}) of 8-17_short (left panels) and full-length bimolecular construct of 8-17 DNAzyme (right panels) as functions of Zn^{2+} concentrations Mg^{2+} and Pb^{2+} (black dots). Fits to a 1:1 interaction model between the DNAzyme and metal ion are depicted as green lines. DNAzyme concentration of 40 µM was used throughout the measurements. Source data are provided as a Source Data file.

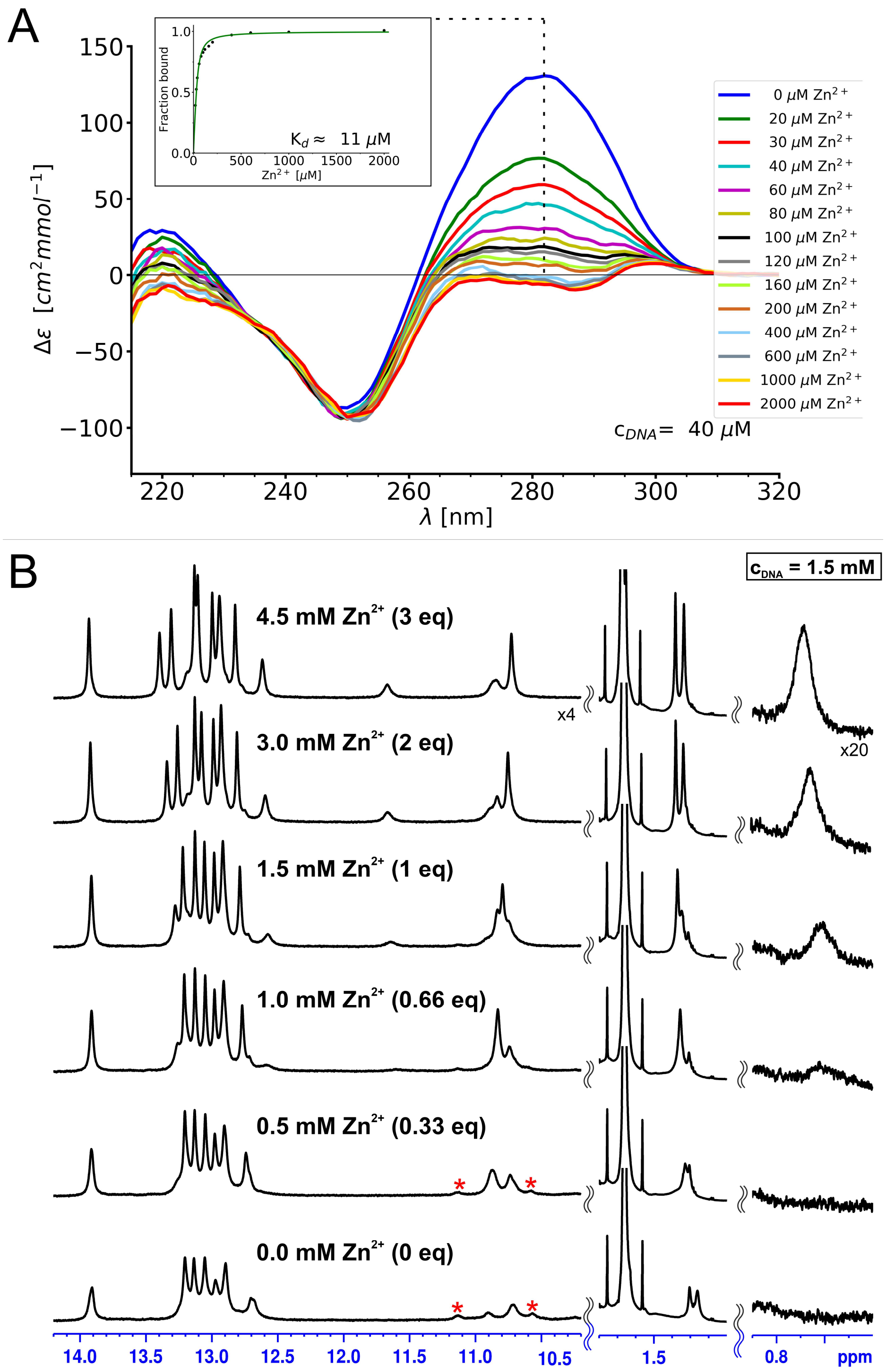

Supplementary Figure 4. Zn^{2+}-induced folding of 8-17_short monitored by a) CD spectroscopy and b) 1D ^{1}H-NMR at pH 7 (compare to pH 6 data of Figure 3). NMR resonances belonging to an initially present second spectral form are marked by red stars. The titration was performed in 10 mM sodium cacodylate buffer which originally contained no additional salts. The strong signal around 1.7 ppm corresponds to the cacodylate methyl resonance. Source data are provided as a Source Data file.

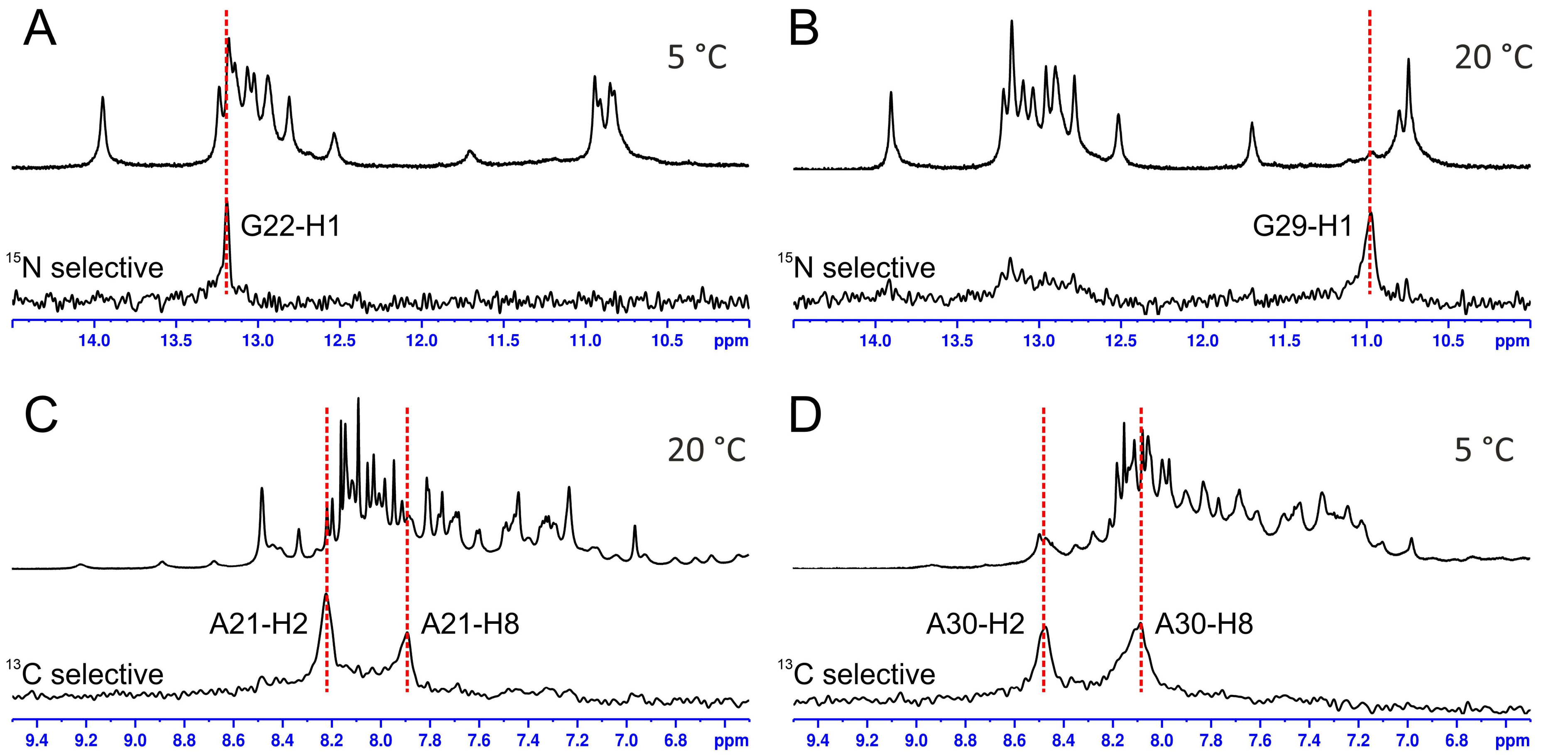

Supplementary Figure 5. The confirmation of spectral assignment of imino and aromatic protons of strictly conserved residues within the 8-17 DNAzyme by the means of site specific ^{13}C-^{15}N-labeling A) G22 imino proton, B) G29 imino proton, C) A21 H2 and H8 protons, C) A30 H2 and H8 protons. In the case of adenosine aromatic protons the h2/H8 distinction was made based on the ^{13}C chemical shift in the HC-HSQC (not shown).

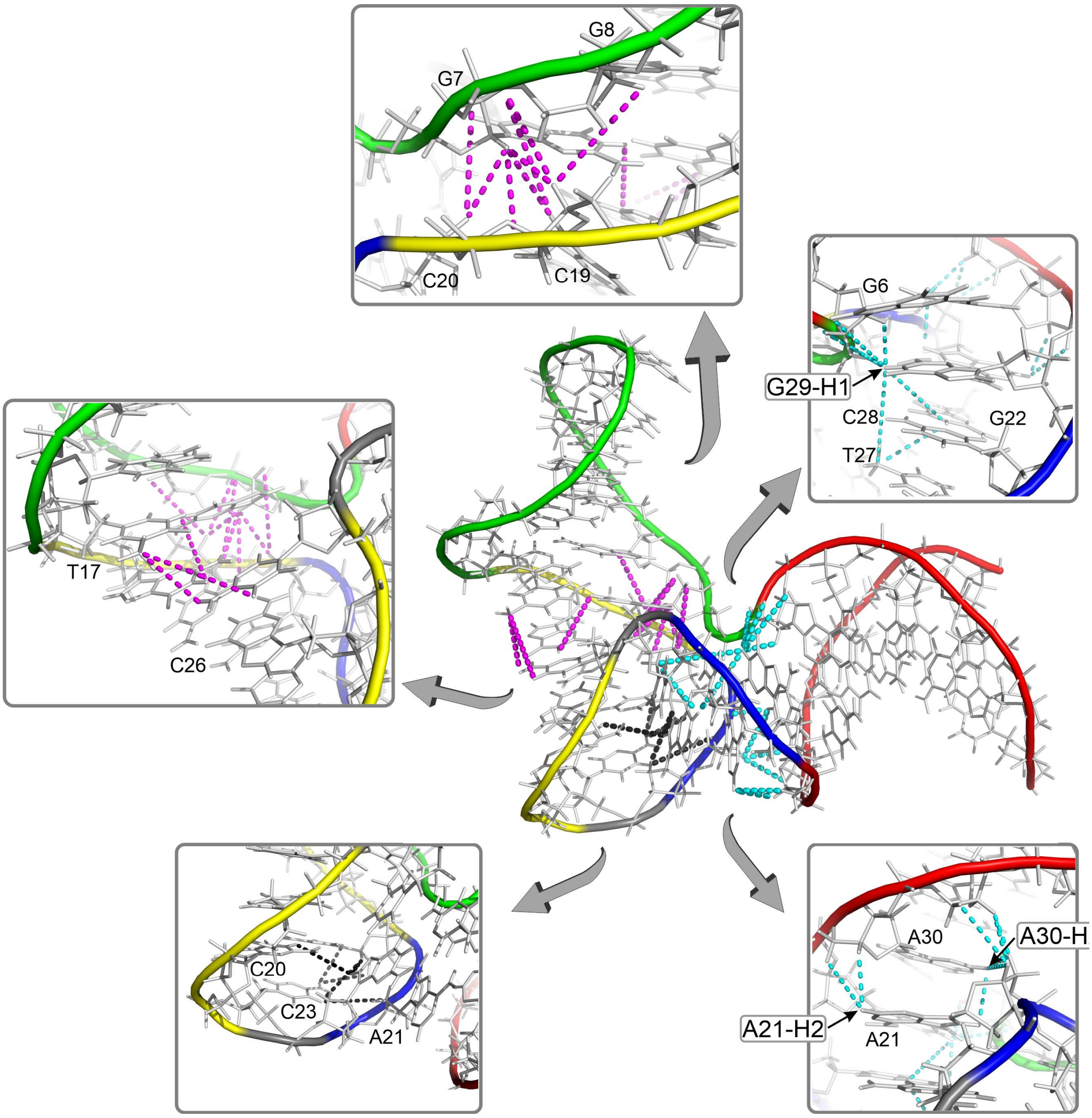

Supplementary Figure 6 The set of long-range NOEs measured within the catalytic domain of 8-17_short visualized on the 3D structure as colored dashes (for a detailed description see SI text).

A)

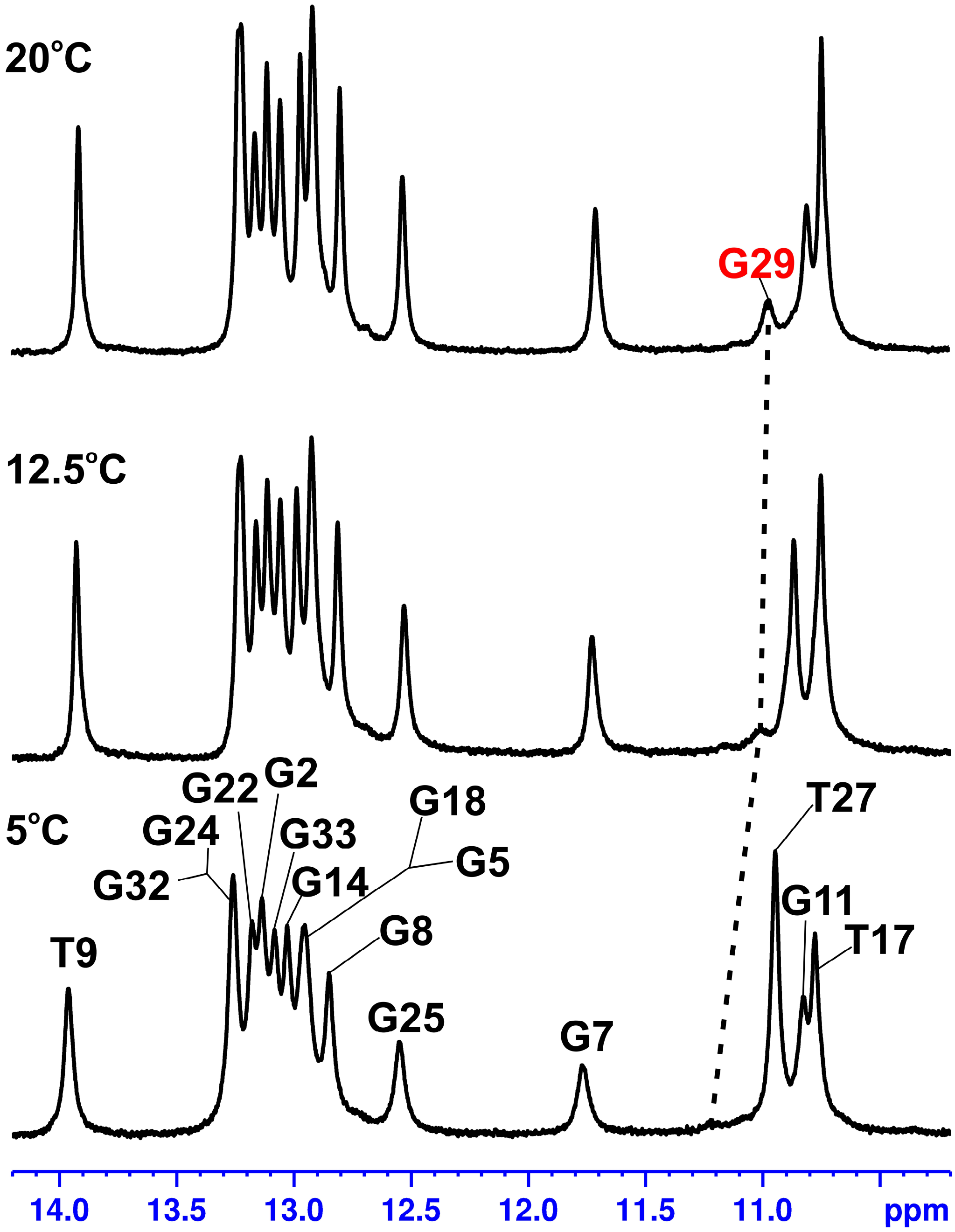

20°C
G29
12.5°C
5°C
T9
G32
G24
G22
G2
G33
G14
G18
G5
G8
G25
G7
T27
G11
T17
14.0
13.5
13.0
12.5
12.0
11.5
11.0
ppm

B)

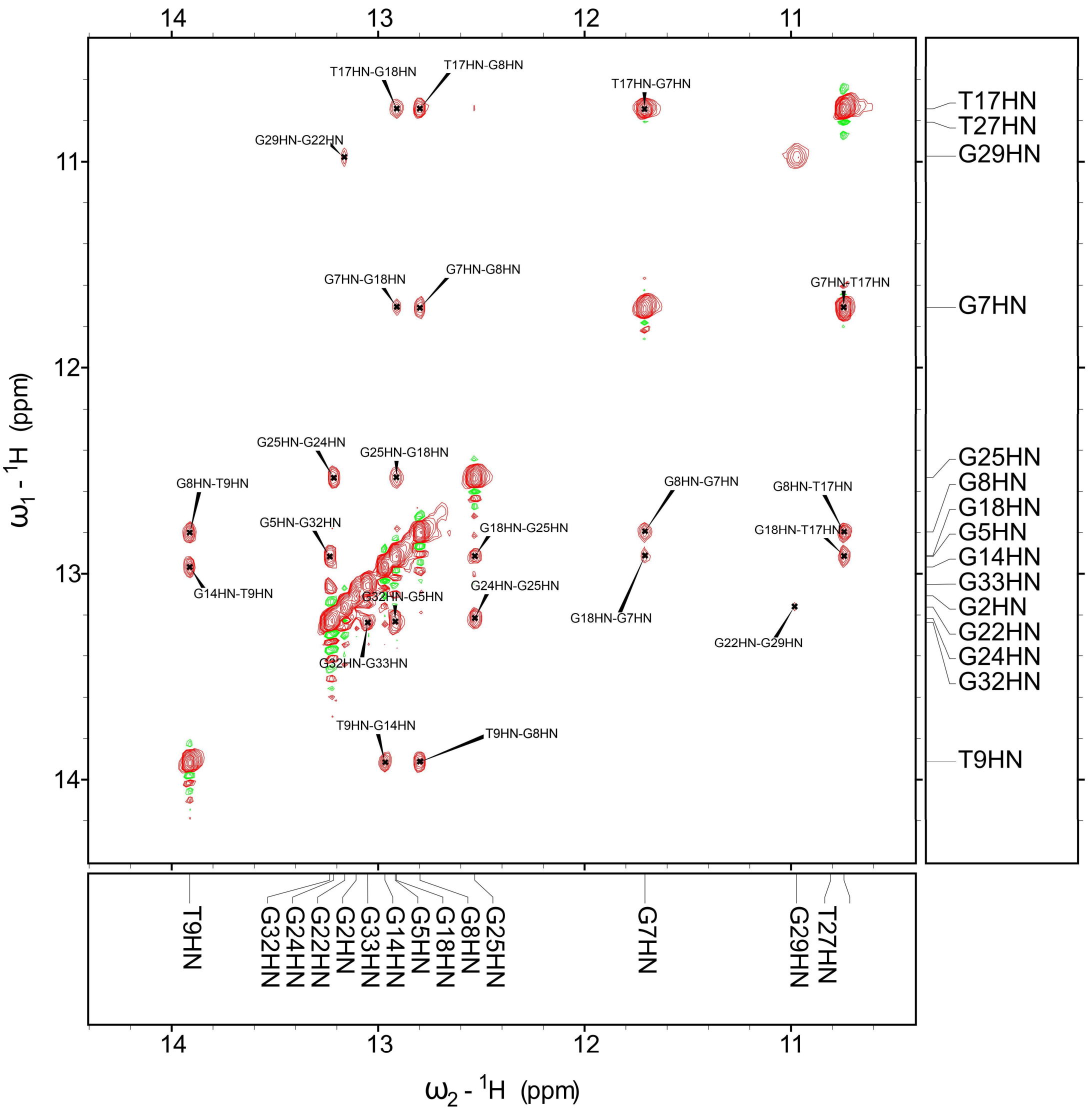

T17HN-G18HN
T17HN-G8HN
T17HN-G7HN
G29HN-G22HN
G7HN-G18HN
G7HN-G8HN
G7HN-T17HN
G25HN-G24HN
G25HN-G18HN
G8HN-T9HN
G5HN-G32HN
G18HN-G25HN
G8HN-G7HN
G8HN-T17HN
G18HN-T17HN
G14HN-T9HN
G32HN-G5HN
G24HN-G25HN
G18HN-G7HN
G22HN-G29HN
G32HN-G33HN
T9HN-G14HN
T9HN-G8HN
T17HN
T27HN
G29HN
G7HN
G25HN
G8HN
G18HN
G5HN
G14HN
G33HN
G2HN
G22HN
G24HN
G32HN
T9HN
ω_1 - ^{1}H (ppm)
ω_2 - ^{1}H (ppm)

C)

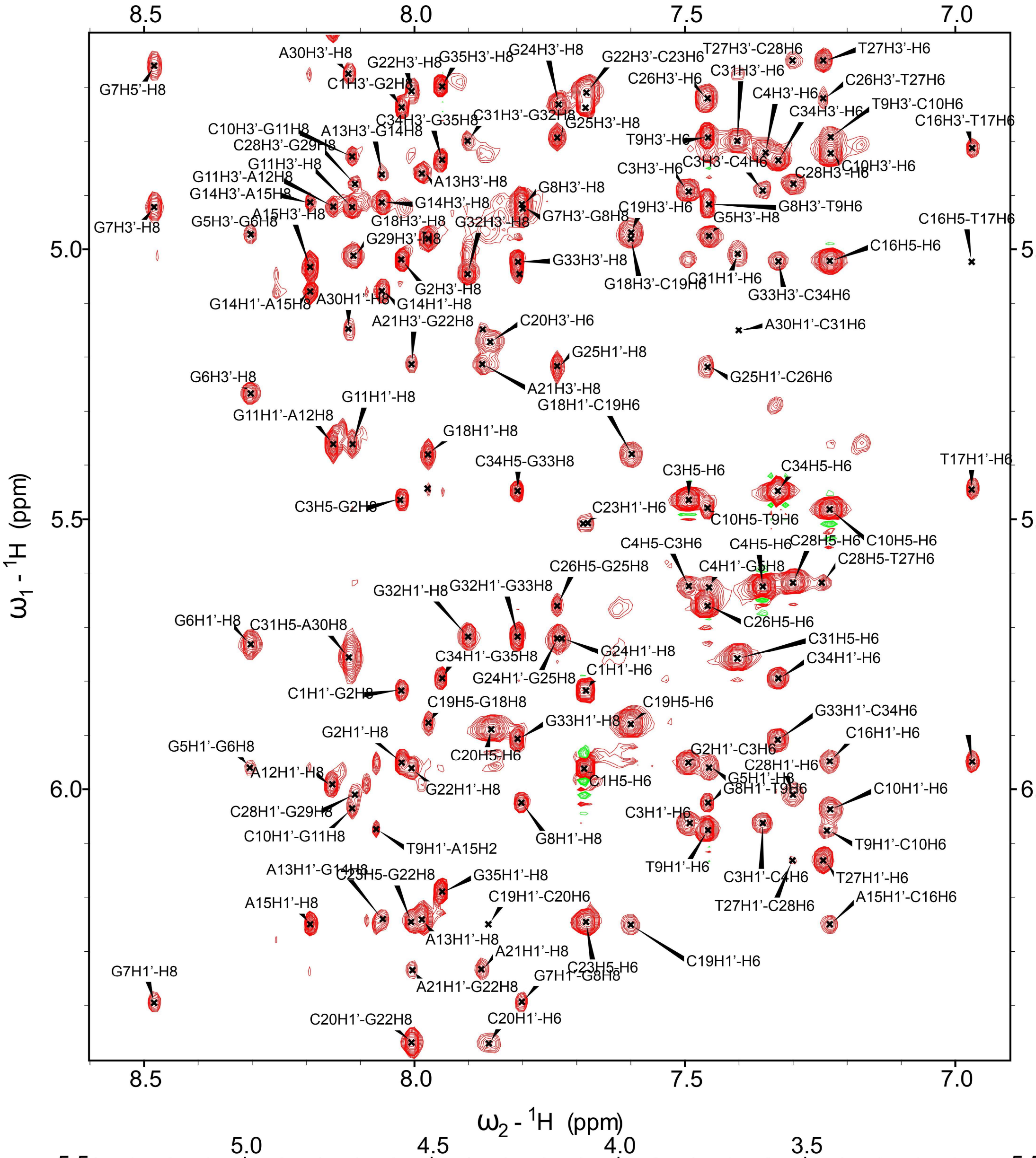

D)

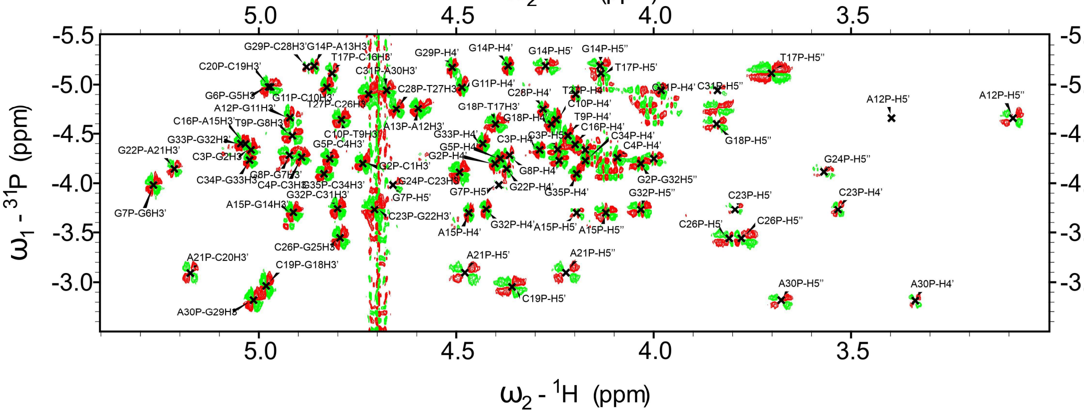

Supplementary Figure 7. Selected fragments of the assigned NMR spectra of 8-17_short in the presence of 2 eq. of Zn^{2+}, as well as, 200 mM of NaCl. A) the imino region of the 1D spectrum at three different temperatures B) the imino-imino region of the ^1H-^1H-NOESY spectrum in H_2O at 20 °C, C) the aromatic-to-anomeric and aromatic-to-H3' regions of the ^1H-^1H-NOESY spectrum in D_2O at 35 °C, D) the ^1H-^{31}P COSY spectrum recorded spectrum in D_2O at 35 °C

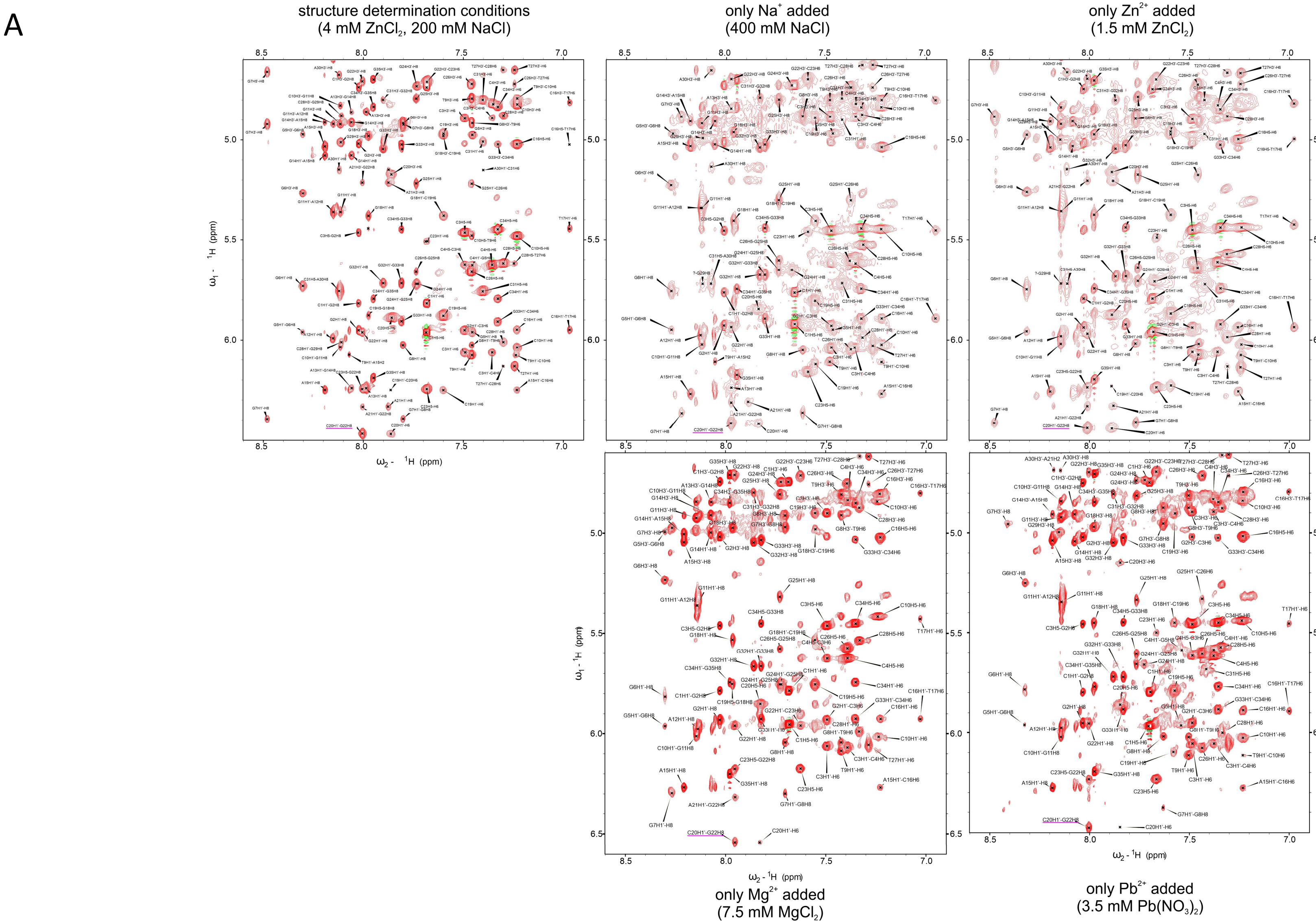
A
structure determination conditions
(4 mM ZnCl$_2$, 200 mM NaCl)
only Na$^+$ added
(400 mM NaCl)
only Zn^{2+} added
(1.5 mM ZnCl$_2$)
only Mg^{2+} added
(7.5 mM MgCl$_2$)
only Pb^{2+} added
(3.5 mM Pb(NO$_3$)$_2$)
ω$_2$ - ^{1}H (ppm)
ω$_1$ - ^{1}H (ppm)
8.5
8.0
7.5
7.0
5.0
5.5
6.0
6.5

B

structure determination conditions (4 mM $ZnCl_2$, 200 mM NaCl)

only Na^+ added (400 mM NaCl)

only Zn^{2+} added (1.5 mM $ZnCl_2$)

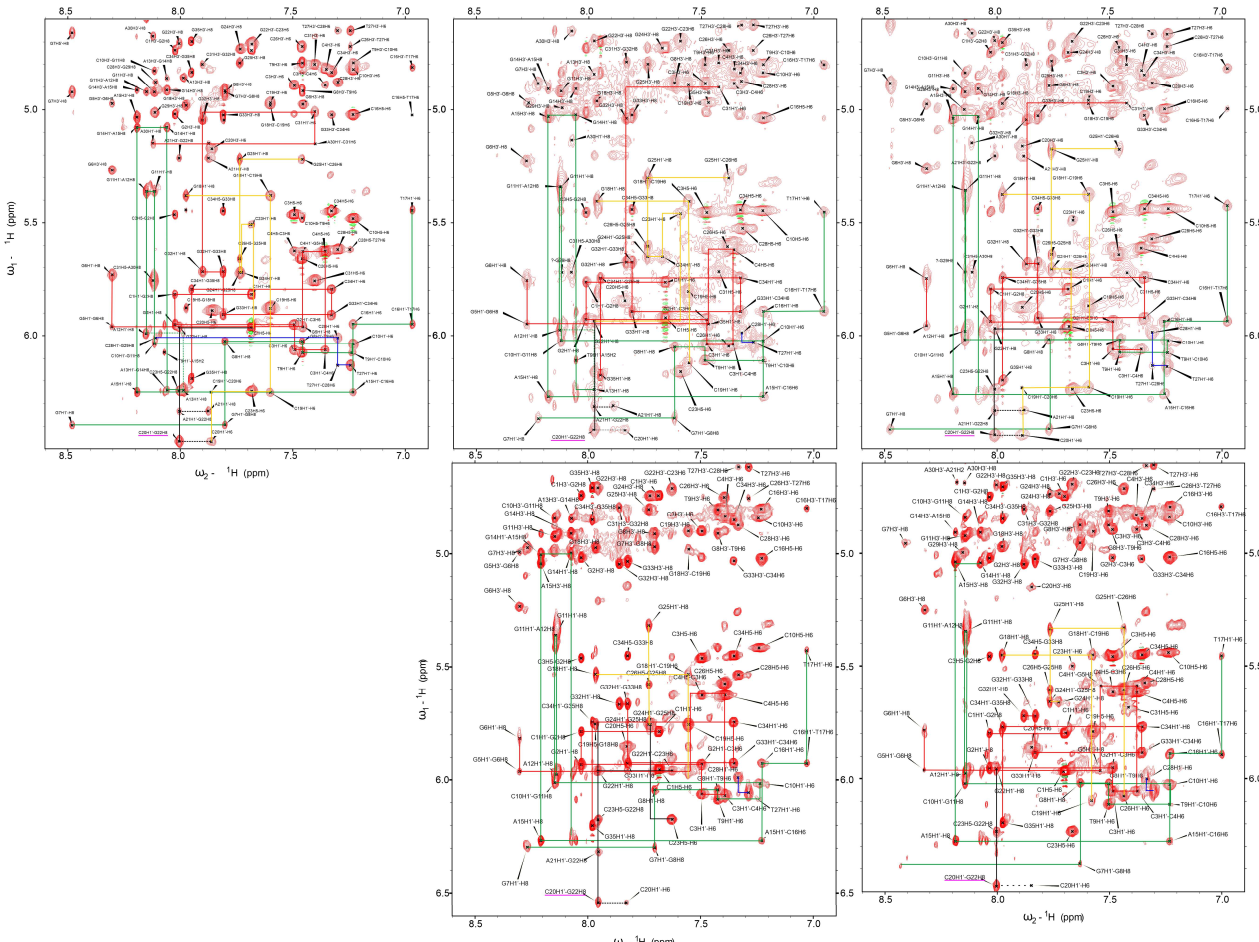

only Mg^{2+} added (7.5 mM $MgCl_2$)

only Pb^{2+} added (3.5 mM $Pb(NO_3)_2$)

C

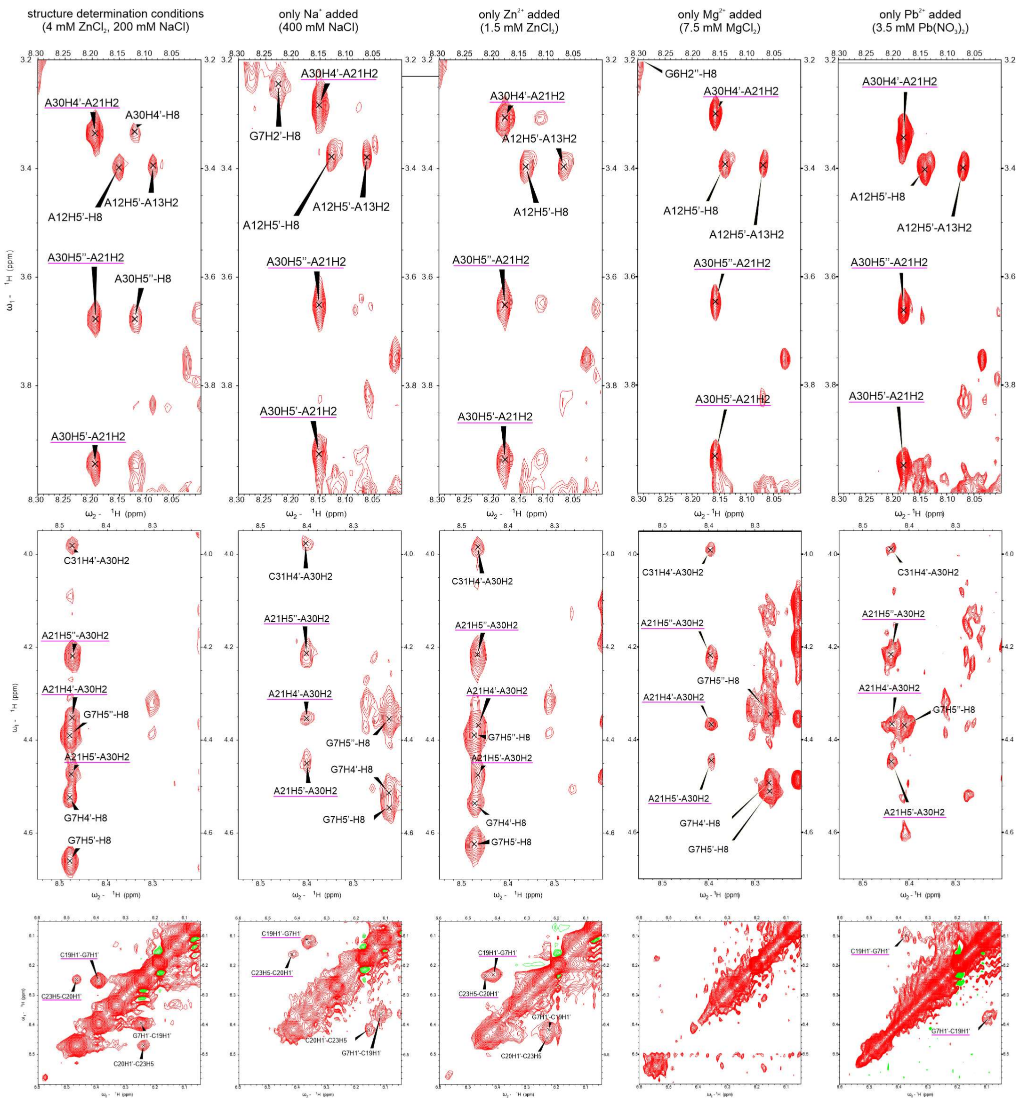

Supplementary Figure 8. Snippets of NOESY spectra of 8-17_short in presence of different metal ions. A) The entire aromatic-to-anomeric and aromatic-to-H3' regions with H1'-H6/H8 NOESY-walks with each helix marked following the color-code of Figure 4, B) Close-ups on regions containing some of the important long-range NOEs. In all panels long range NOEs in the catalytic domain are underscored with a purple line.

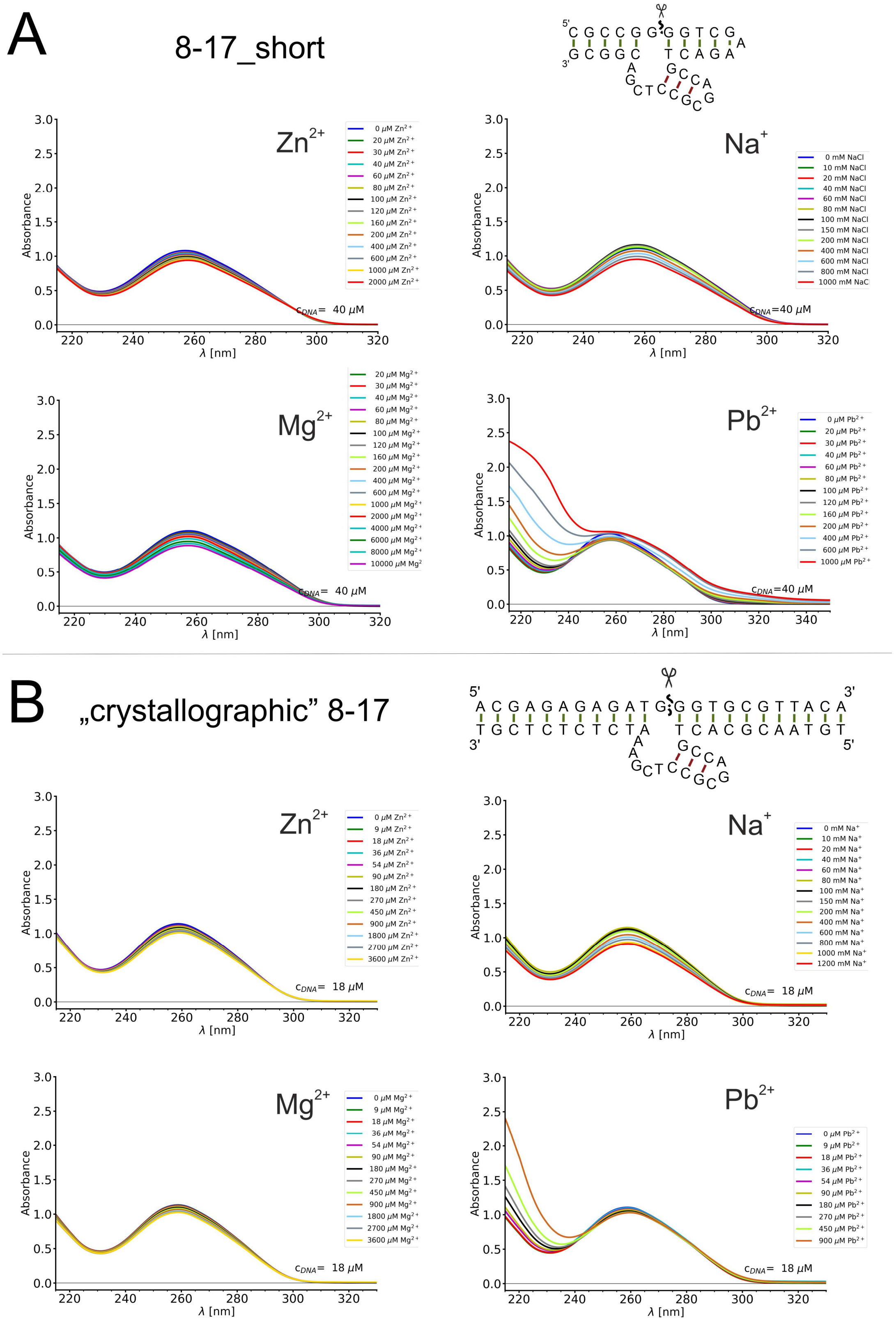

Supplementary Figure 9 The UV spectra recorded during metal ion titrations of DNAzyme 8-17 constructs A) 8-17_short and B) full-length bimolecular “crystallographic” construct. Source data are provided as a Source Data file.

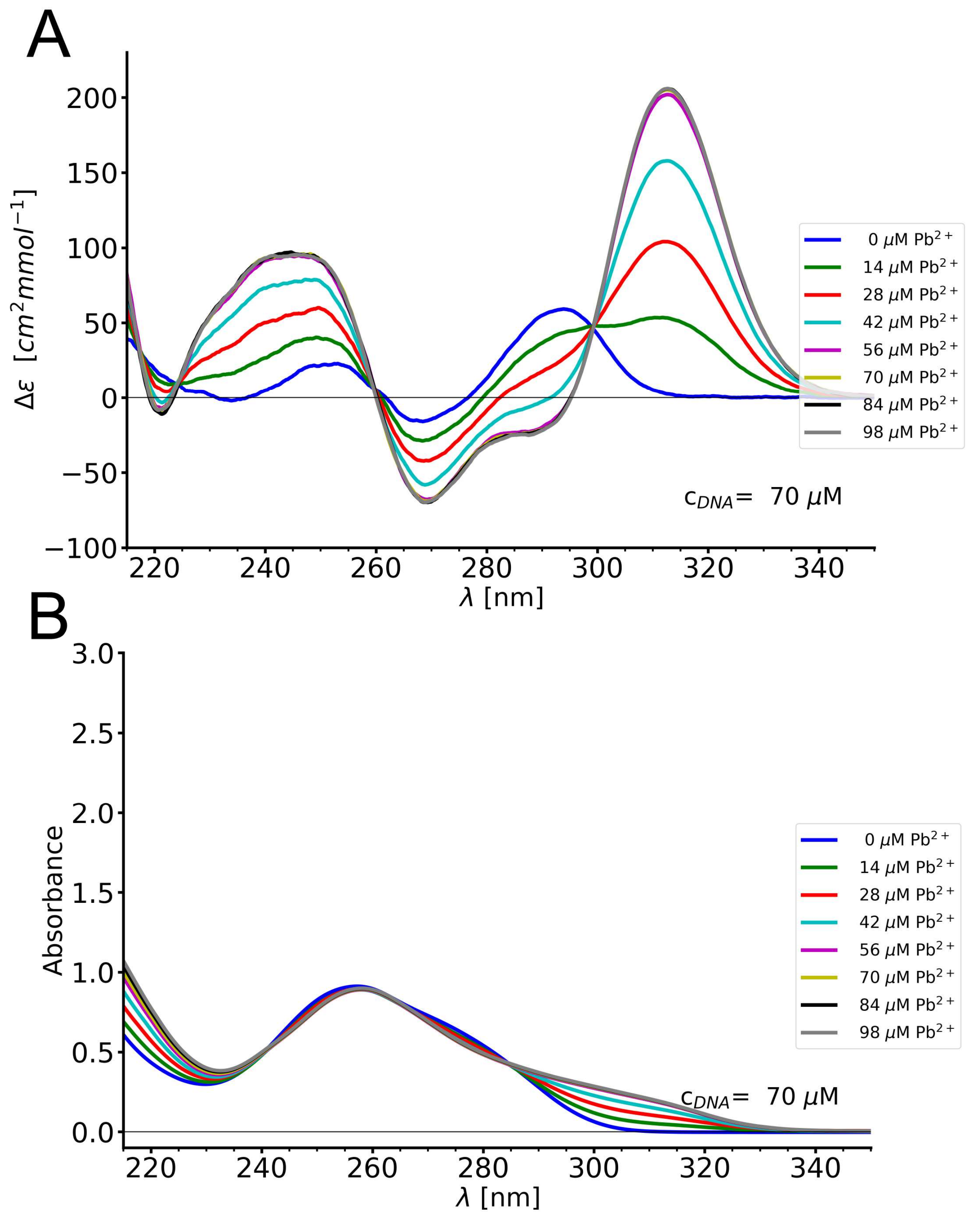

Supplementary Figure 10 The CD (A) and UV (B) UV spectra of thrombin binding aptamer (TBA) recorded during a Pb^{2+} titration. Source data are provided as a Source Data file.

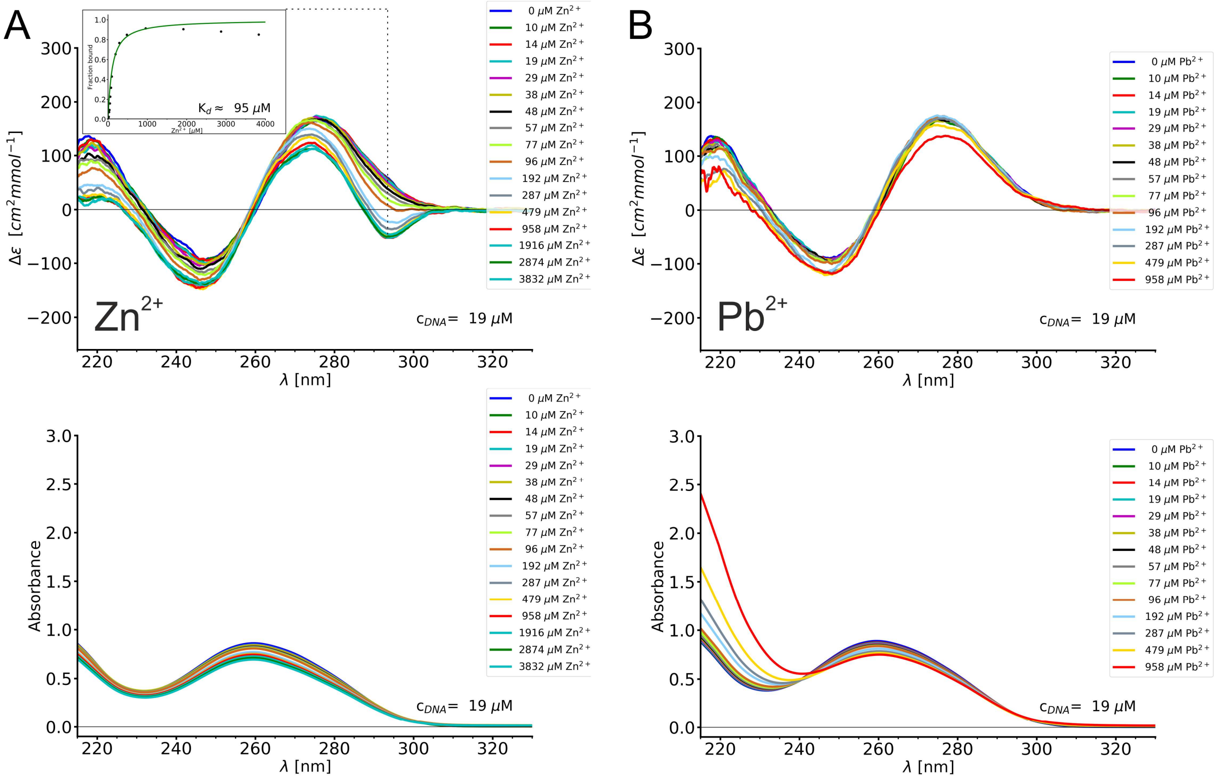

Supplementary Figure 11 The CD (top) and UV (bottom) spectra recorded for the 8-17 DNAzyme construct used in the previous CD investigation[1] a) Zn^{2+} titration b) Pb^{2+} titration. Source data are provided as a Source Data file.

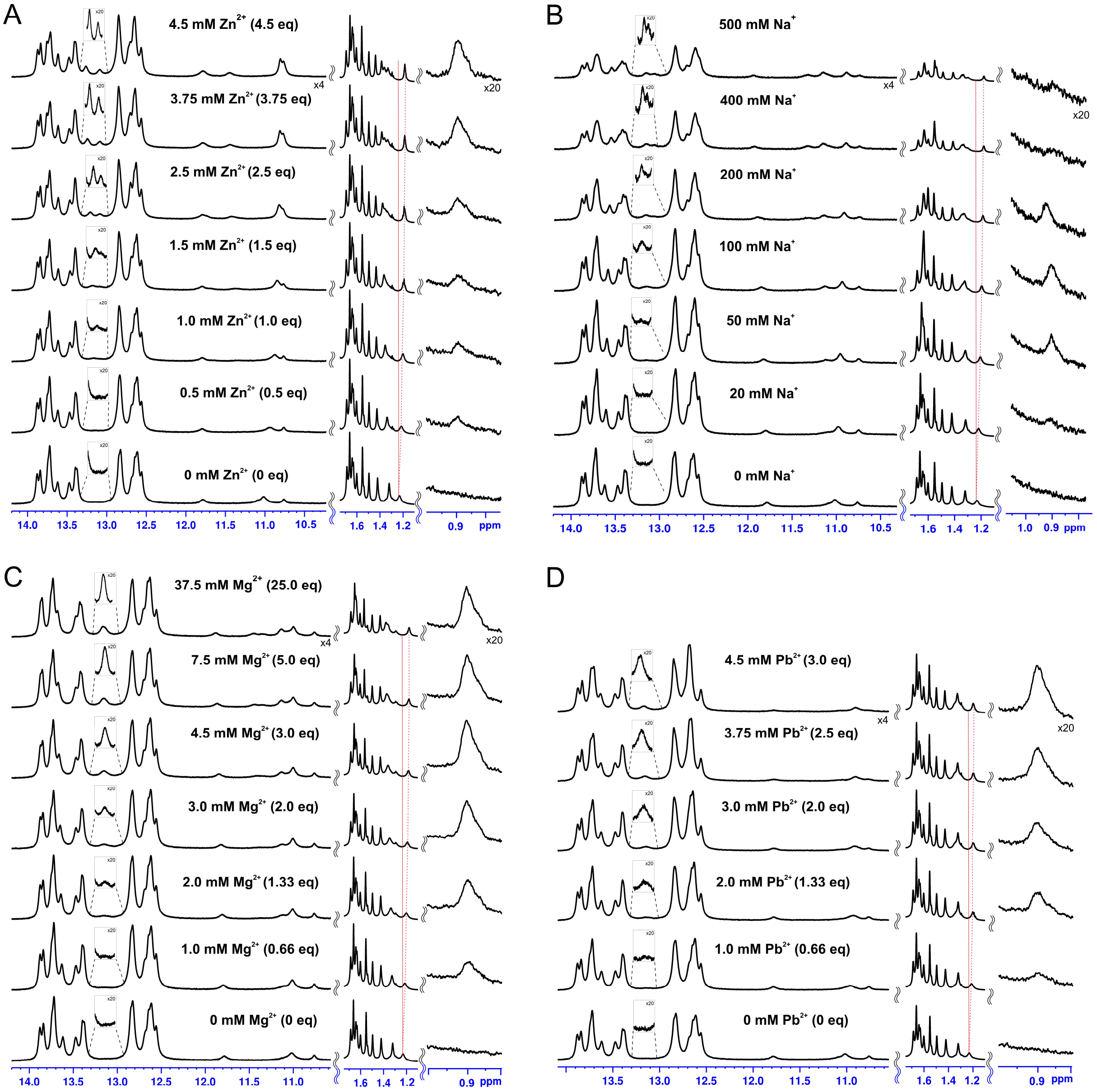

Supplementary Figure 12 1D NMR-monitored titrations of the 8-17 DNAzyme construct used in the previous crystallographic investigation (sequence in Figure 1b) with four different metal ions: a) Zn^{2+}, b) Na^{+}, c) Mg^{2+} and d) Pb^{2+}.

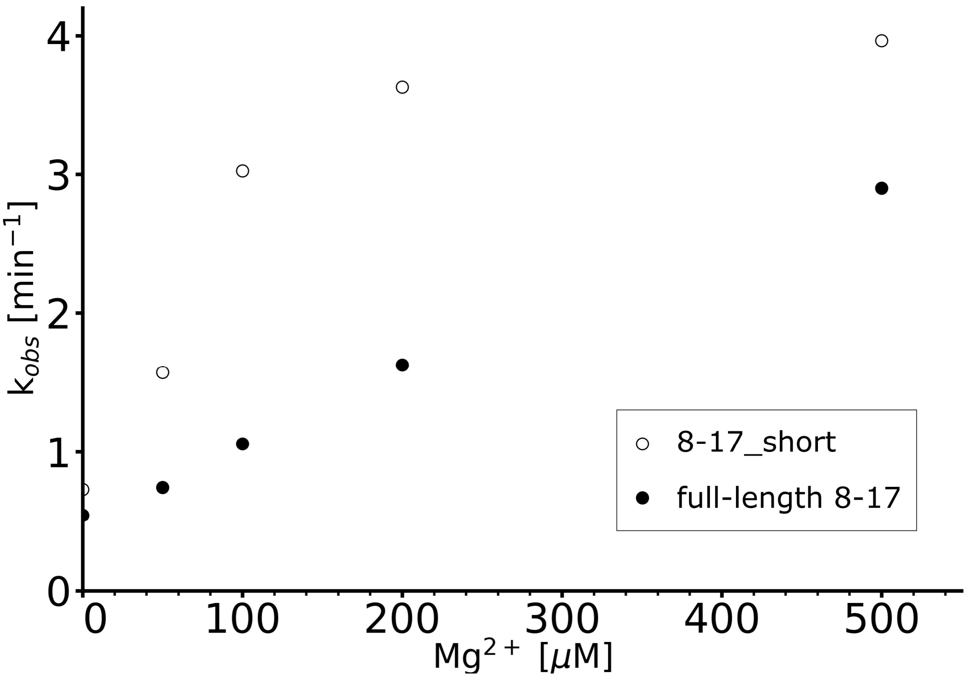

Supplementary Figure 13. The catalytic activity of two 8-17 DNAzyme variants in presence of 50 µM Pb^{2+} and varying concentrations of Mg^{2+}. DNAzyme concentration of 40 µM was used. Source data are provided as a Source Data file.

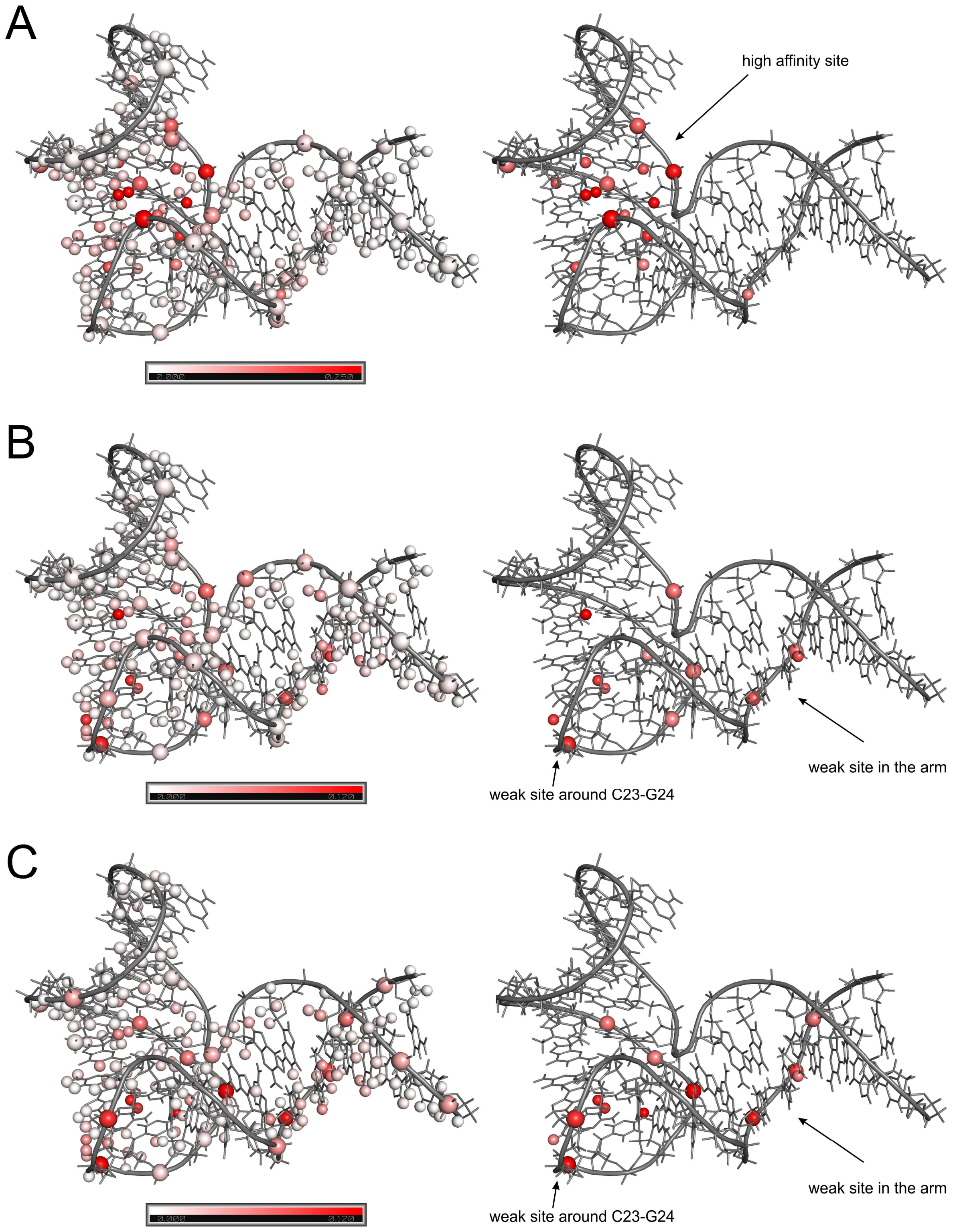

Supplementary Figure 14. Chemical shift perturbations (CSPs) experienced by 8-17_short upon Zn^{2+} titration. A) CSPs induced by 1 equivalent of Zn^{2+} titrated into a sample structured by 400 mM of NaCl, B) additional CSPs induced by 2 more equivalents of Zn^{2+} titrated into the same sample, C) CSPs induced by 2 additional equivalents of Zn^{2+} titrated into a sample structured by 1.5 mM of Zn^{2+} (no NaCl present). In each panel the left side shows all the measured CSPs, while the right one only the most pronounced ones (> 0.1 ppm for panel A, and above 0.05 ppm for panels B and C). All CSPs are color coded from 0.00 [ppm] (white) to ≥ 0.25 [ppm] (red) for panel A, and from 0.00 [ppm] (white) to ≥ 0.12 [ppm] (red) for panels B and C. Source data are provided as Supplementary Data files.

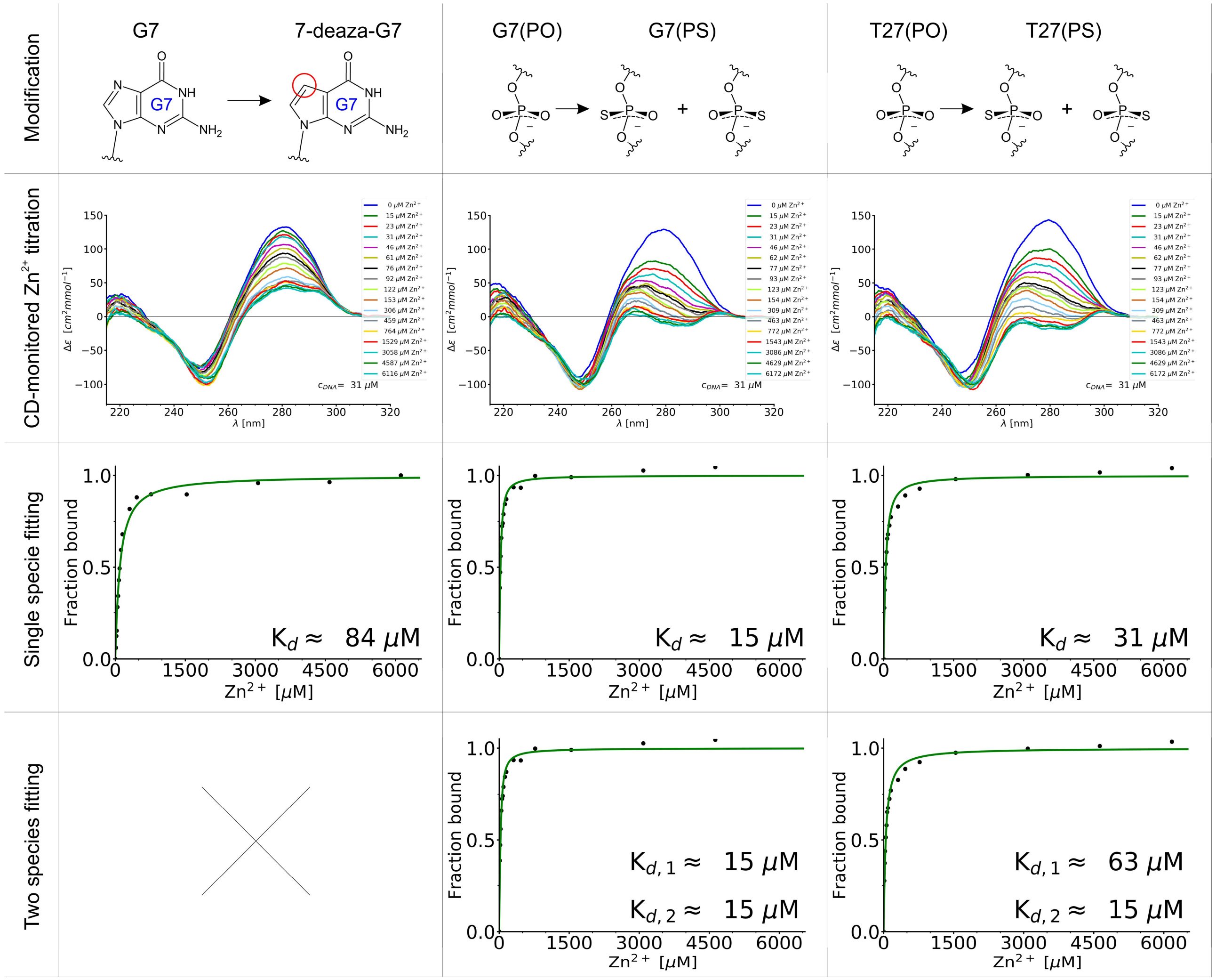

Supplementary Figure 15. CD-monitored Zn^{2+} titrations of mutated variants of 8-17_short containing either the 7-deaza-dG at position G7 or the phosphorothioate (PS) modification at positions G7 or T27. The fitting procedure for the PS-modified variants is described in the SI text. Source data are provided as a Source Data file.

Supplementary Table 1. Structure determination statistics for 8-17 DNAzyme.

	Structure name<br>8OR8
**NMR distance and dihedral constraints**	
Distance restraints	
Total NOE	365
Intra-residue	160
Inter-residue	205
Sequential ($\|i-j\| = 1$)	163
Nonsequential ($\|i-j\| > 1$)	42
Hydrogen bonds	80*
Total dihedral angle restraints	156
Base pair	35
Sugar pucker	23
Backbone	98
Based on B-form geometry	0
**Structure statistics**	
Violations (mean and s.d.)	
Distance constraints (Å)	0.02 (0.00)
Dihedral angle constraints (º)	0.9 (0.7)
Max. dihedral angle violation (º)	23.7
Max. distance constraint violation (Å)	0.36
Deviations from idealized geometry	
Bond lengths (Å)	0.01 (0.00)
Bond angles (º)	1.65 (0.02)
Impropers (º)	2.46 (0.19)
Average pairwise r.m.s. deviation** (Å)	
All heavy atoms	1.83 (0.47)
Catalytic domain (G18-A30) heavy atoms	0.99 (0.22)

*40 hydrogen bonds imposed, with two restraints per bond (heavy atom-heavy atom and hydrogen-heavy atom)
**Pairwise r.m.s. deviation was calculated among 20 refined structures.

Supplementary Table 2 Long-range NOE restraints involving residues of the catalytic domain.

Atoms involved		Maximum allowed distance	Distance range within the NMR ensemble	Observability in other conditions			
				400 mM Na^{+}	1 eq Zn^{2+} (1.5 mM)	5 eq Mg^{2+} (7.5 mM)	2.33 eq Pb2+ (3.5 mM)
NOEs reporting on the geometry of the junction between Helices 2 and 3							
G7(**N1**)-H1'	C19(**N4**)-H1'	5	2.50-4.69	present	present	uncertain[a]	present
G7(N1)-H2''	C20(N5)-H5''	5	2.43-5.42	uncertain[b]	present	uncertain[c]	not observed
T17(T2)-Me	C26(N11)-H5	6	4.73-5.63	present	present	not observed	present
C19(N4)-H2''	G7(N1)-H1'	5	2.77-4.39	present	present	uncertain[b]	uncertain[b]
C19(N4)-H4'	G7(N1)-H1'	5	2.07-2.50	present	present	uncertain[a]	uncertain[a]
C19(N4)-H4'	G7(N1)-H2'	5	2.61-4.73	uncertain[b]	present	uncertain[a]	uncertain[a]
C19(N4)-H4'	G7(N1)-H2''	5	2.61-4.73	uncertain[b]	present	uncertain[a]	uncertain[a]
C20(N5)-H5''	G7(N1)-H1'	5	2.22-4.16	present	present	uncertain[c]	present
G7(N1)-H2''	C19(N4)-H1'	7	4.16-6.14	uncertain[b]	uncertain[b]	uncertain[a]	uncertain[a]
C19(N4)-H4'	G8(--)-H1'	7	3.97-5.66	present	present	uncertain[a]	uncertain[a]
G7(N1)-H1	G18(N3)-H1	6.5	4.82-5.80	present	present	uncertain[a]	uncertain[a]
T17(T2)-Me	C26(N11)-H42	6	3.47-3.79	present	present	present	present
NOEs fixing the geometry of Helix 4 as well as its relation to Helix 1 and unpaired T27(**N12**)							
A21(A6)-H2	A30(N15)-H4'	5	2.62-2.93	present	present	present	present
A30(N15)-H2	A21(A6)-H8	5	2.65-2.65	present	present	uncertain[a]	present
A21(A6)-H5'	A30(N15)-H2	5	2.32-3.17	present	present	present	present
A21(A6)-H5''	A30(N15)-H2	5	2.32-3.17	present	present	present	present
A30(N15)-H5'	A21(A6)-H2	5	2.74-3.83	present	present	present	present
A30(N15)-H5''	A21(A6)-H2	5	2.74-3.83	present	present	present	present
A21(A6)-H4'	A30(N15)-H2	7	3.84-4.60	present	present[c]	present	present
A21(A6)-H5'	C31(--)-H4'	7	2.30-3.01	uncertain[c]	uncertain[c]	uncertain[c]	uncertain[c]
C31(--)-H4'	A21(A6)-H4'	7	2.35-2.53	uncertain[c]	uncertain[a]	uncertain[c]	uncertain[c]
C31(--)-H5''	A21(A6)-H4'	7	2.45-4.40	uncertain[a]	uncertain[a]	uncertain[a]	uncertain[a]
G29(G14)-H1	G22(G7)-H1	6.5	4.99-5.02	uncertain[a]	uncertain[a]	uncertain[a]	uncertain[a]
G6(N18)-H1'	G29(G14)-H1	6.5	2.43-2.78	uncertain[a]	uncertain[a]	uncertain[a]	uncertain[a]

G6(N18)-H2'	G29(G14)-H1	6.5	2.08-2.32	uncertain[a]	uncertain[a]	uncertain[a]	uncertain[a]
G6(N18)-H3'	G29(G14)-H1	6.5	3.92-4.25	uncertain[a]	uncertain[a]	uncertain[a]	uncertain[a]
G6(N18)-H8	G29(G14)-H1	6.5	4.98-5.03	uncertain[a]	uncertain[a]	uncertain[a]	uncertain[a]
T27(N12)-Me	G29(G14)-H1	7.5	5.91-5.91	uncertain[a]	uncertain[a]	uncertain[a]	uncertain[a]
T27(N12)-Me	G22(G7)-H1	5	3.46-4.79	present	present	present	present
NOEs defining the geometry of triloop A21-G22-C23 (**A6-G7-Y8**) with respect to Helix 3							
C20(N5)-H1'	G22(G7)-H8	4	2.06-2.49	present	present	present	present
C20(N5)-H1'	C23(Y8)-H5	5	3.43-4.77	present	present[c]	uncertain[b]	uncertain[b]
A21(A6)-H3'	C23(Y8)-H5	7	4.43-5.23	uncertain[b]	uncertain[b]	uncertain[b]	uncertain[b]
C20(N5)-H2'	C23(Y8)-H41	6.5	2.60-3.69	present	uncertain[c]	uncertain[a]	uncertain[a]
C20(N5)-H6	C23(Y8)-H41	6.5	3.59-3.60	not observed	uncertain[c]	uncertain[a]	uncertain[a]
G24(N9)-H1	G22(G7)-H8	5	4.26-4.92	present	present	present	present

[a]one of the atoms not assigned in these conditions
[b]very broad/weak peak barely above noise level
[c]affected by spectral overlap

Supplementary Note 1. 8-17 DNAzyme construct optimization for NMR studies

Most commonly used constructs of the 8-17 DNAzyme contain substrate binding arms of 10-12 nucleotides each, which translates to the entire DNAzyme-substrate complex being composed of around 60 nucleotides. A structural study of a DNA molecule of this size by solution NMR without isotope enrichment would be significantly burdened by excessive spectral overlap and broad resonance lines. To enable an NMR study of the 8-17 DNAzyme we have thus embarked on a systematic search for an 8-17 construct of a significantly reduced size retaining the catalytic activity, concentrating mainly on the substrate binding arms, as within the catalytic domain only a single residue can be safely truncated. Simple truncation of the arms turned out not to be most efficient approach, as already for constructs with the arms shortened to 6 nt each, the DNAzyme strand ceased to quantitatively hybridize with the substrate strand at room temperature (data not shown). Thus, in order to reduce the entropic cost of DNAzyme-substrate hybridization we turned to unimolecular constructs by connecting the two strands with a trinucleotide (GAA) loop capping one of the arms (Supplementary Figure 1). To identify the ideal arm lengths and sequences within such a framework, we have systematically evaluated in silico (using the UnaFold software[2]) 1) the thermodynamic stability and 2) the potential to form unwanted alternative secondary structures, for each possible 8-17 construct with arm lengths of 5-5, 5-4, 5-3, 6-2 and 4-4 (the numbers refer to the lengths of the "free" and "capped" arms, respectively). Around a dozen such constructs with the best predicted stabilities and without predicted alternative secondary structures were then chemically synthesized and evaluated by NMR for 1) monodispersity in presence of Zn^{2+} ions and 2) thermal stability of the folded structure in the same conditions. The results obtained for each construct are summarized in Supplementary Figure 1. Among the constructs displaying a single spectral form in the presence of Zn^{2+}, "5-3GTC" (referred to as "8-17_short" from now on) was selected for further studies due to its superior thermal stability, as well as very limited spectral overlap in preliminary 2D NMR spectra (not shown). The selected 8-17_short construct was used throughout the structural studies in its all-DNA noncleavable form. A possible alternative would be to include a 2'-OMe or 2'-F modified nucleotide at the cleavage site to conserve the sugar C3'- conformation, likely adopted by the RNA nucleotide in an active construct. However, the previous crystallographic study[3] reported the structures of both the all-DNA and 2'-OMe modified versions of their 8-17 DNAzyme construct (PDB: 5XM9 and 5XMA, respectively) and found little differences between them.

Supplementary Note 2. NMR spectral patterns observed for the 8-17 short in the structure determination conditions - long-range NOE contacts and uncommon chemical shift values

The sample of 2.2 mM DNAzyme 8-17_short containing 4 mM $ZnCl_2$ and 200mM NaCl yielded very good quality 2D NOESY spectra in the range of temperatures from 5 to 35 °C. The analysis of these spectra allowed us to trace the inter-residue connectivity patterns (*"NOESY-walks"*) for all residues forming the substrate binding arms. The hydrogen atoms belonging to these regions of the molecule produced a standard set of NOE cross-peaks characteristic to B-DNA helices (e. g. H1'-H8/H6 and H3'-H8/H6 NOESY walks visible in Supplementary Figure 7c, first one also traced in Supplementary Figure 8b). The formation of the G7:T17 base pair at the end of Helix 2 was confirmed by the observation of a strong cross-peak between the imino protons of these two residues (Supplementary Figure 7b). The formation of Helix 3 within the catalytic domain was also confirmed by the observation of imino sharp resonances of residues G18, G24 and G25 (Supplementary Figure 7a and b) in G-C base pairs with C26, C19 and C18, respectively. The same conclusion was corroborated by the observation of standard helical NOE contacts between non-exchangeable protons of residues G18-C19-C20 and G24-G25-C26 (traced in Supplementary Figure 8b). NOE cross peaks corresponding to a standard helical "NOESY

walk" were also observed between residues T17 and G18 suggesting coaxial stacking of Helices 2 and 3. On top of the imino resonances belonging to the three expected helices, an additional sharp imino resonance was also observed at 13.16 ppm (at 20 °C). It belonged to a guanosine residue (as confirmed by ^{1}H-^{15}N HSQC experiment, not shown) forming a G:C base pair with C28 (as confirmed by characteristic strong NOE cross peaks to the NH2 protons of the said cytidine). This unexpected imino resonance was assigned to G22 using site specific ^{15}N-labeling of this position (Supplementary Figure 5a) and thus the additional base pair turned out to be G22:C28. This is the same base pair that was previously observed in the apo and Pb^{2+}-bound crystal structures (**G7:C13** in the standard 8-17 DNAzyme numbering scheme). On top of this additional base pairing, an exceptionally rich set of long-range NOEs was observed for residues belonging to the catalytic domain counting 35 long-range NOEs in total (Table S2).

While this classification only became apparent after solving the 3D structure, these NOEs can be divided into three main groups based on their position within the tertiary fold of the catalytic domain (Table S2). The first group of NOEs arises due to coaxial stacking of Helices 2 and 3 in a geometry which brings the sugar moiety of G7 (from terminal G:T pair of Helix 2) very close to the corresponding moieties of residues C19 and C20 (from Helix 3). Two NOEs between atoms of residues T17 (Helix 2) and C26 (Helix 3) also belong to this group. The second group of NOEs define the geometry of an additional Helix 4 (see main text) which contained the abovementioned G22:C28 base pair, as well as its position relative to Helix 1. These NOEs can be further subdivided into two regions 1) 8 long-range NOEs are observed between atoms belonging to residues A21 and A30 (or A21 and C31) and 2) the imino proton of G29 gives rise to a total of 6 long-range NOEs to protons belonging to residues G6, G22 and T27. Regarding the first sub-group multiple of those NOEs involve aromatic H2 protons of either A21 or A30. As H2 protons in non-standard structural elements can be notoriously difficult to assign, the resonance assignments of both A21-H2 and A30-H2 were confirmed through site specific ^{13}C-labeling (Supplementary Figure 5 c and d). The second sub group of NOEs all belonged to G29-H1 (imino) proton and thus the assignment of this proton was also confirmed through site specific ^{15}N-labeling (Supplementary Figure 5b). It has to be noted that G29-H1 is not involved in base pairing interactions in the 3D structure (indeed it is the imino proton of the general base - **G14** in the standard 8-17 DNAzyme numbering scheme) and as such this resonance it much broader than most other imino resonances. It is the sharpest at 20 °C (Supplementary Figure 7a) and thus the NOE restraints involving it were all derived from a NOESY spectrum measured at this temperature. The third major group of non-sequential NOE contacts define the geometry of the triloop A21-G22-C23 which caps Helix 3. As the residues A21 and G22 participate in the formation of Helix 4 these NOEs can also be seen as informing on the mutual positions of Helices 3 and 4.

While the set of long-range NOEs discussed above (together with much more numerous sets of sequential and intra-residue NOEs, complemented by torsion angle restraints, Table S1) is what allowed us to solve the 3D structure of the DNAzyme, several non-standard values of chemical shifts were also observed throughout the molecule and are worth discussing. Coaxial stacking of Helices 2 and 3 places the sugar moiety of T17 (Helix 2) directly in front of the aromatic ring of G18 (Helix 3). Such a position above an aromatic ring is known to produce strong shielding effects in DNA sugar protons (e.g. [4]). Indeed, the T17-H2' proton positioned closest to G18 aromatic ring is strongly upfield shifted to 0.615 ppm. This is over three standard deviations away the average chemical shift for thymidine H2' equal to 2.126 ± 0.428 ppm according to Biological Magnetic Resonance Data Bank (BMRB) chemical shifts statistics (https://bmrb.io/ref_info/csstats.php). While the H2' proton is the most affected, the same effect also manifests itself for other T17 sugar protons all of which – with the exception of H4' – have chemical shifts significantly below the BMRB averages.

A similar situation is also observed on the junction between Helices 1 and 4 – the sugar protons of A30 (Helix 4) are all upfield shifted compared to BMRB averages with the most affected being H2' proton (at 1.424 ppm; BMRB average 2.616 ± 0.381 ppm) and H4' (at 3.335 ppm; BMRB average 4.288 ± 0.423 ppm). In this case the distorted helical geometry of the short Helix 4 also comes to play and the analysis of the 3D structure reveals that A30 sugar protons may fall within the shielding cones of both C31 (Helix 1) and G29 (Helix 4).

Supplementary Note 3. NMR observations for samples containing only a single metal ion type (Na^+, Mg^{2+}, Zn^{2+} or Pb^{2+})

As described in the main text, folding transitions in DNAzyme 8-17_short were induced by titration with either Na^+ (Figure 6a), Mg^{2+} (Figure 6b), Zn^{2+} (Figure 3b), or Pb^{2+} (Figure 7b). These transitions all saturated at different numbers of equivalents of the titrated ion, correlating with their different binding affinities estimated using CD spectroscopy. What can however be said about the structures of the products of each transition and their similarities (or dissimilarities) to the structure solved with high resolution in presence of 4 mM Zn^{2+} and 200 mM NaCl? To study each structure in depth standard sets of 2D NMR spectra were recorded for each sample, as was done before in the original conditions containing a Zn^{2+}/Na^+ mixture and described in the Methods section. While all four new samples gave reasonably well resolved 2D data (see Supplementary Figure 8a) up to around 20 °C, their quality was clearly inferior to those obtained in the original conditions. Among the four samples relatively the sharpest spectral lines were seen in the presence of Zn^{2+}, followed by Na^+, Pb^{2+} and finally Mg^{2+}. The presence of broader peaks increased spectral overlap, made integration uncertain in many cases, as well as meant that many weaker NOE cross-peaks were lost in the noise.

For these reasons we decided not to attempt independent structure determination calculations in each of the new conditions. Instead, we collected as exhaustive as possible sets of 1) chemical shift assignments and 2) NOE cross-peaks for each sample to be compared with those recorded in the original structure determination conditions. As the pattern of NOE cross peaks is what defines the 3D fold determined by NMR, a perfect overlap between NOEs collected in one of the new conditions with those from the original sample would constitute a sufficient proof of the same DNAzyme fold being present in the two samples. As however it was clear that the presence of many weaker NOEs from the original conditions would not be possible to verify due to inferior signal-to-noise ratios in the new samples, we also decided to compare chemical shift values measured with different metal ions being present. While NMR chemical shift values are usually not directly used for structure determination in DNA, they are sensitive reporters of the 3D environment of each proton in a DNA structure and a perfectly repeating pattern of chemical shifts would also be sufficient to deem two structures identical. Regarding the NOE set comparisons all four samples generally displayed pathways of sequential NOE connectivities very similar to what was observed in the original conditions (see for example NOESY-walks depicted in Supplementary Figure 8a). However, when comparing NOE sets it is worth to concentrate on the long-range NOEs observed within the catalytic domain (Table S2, Supplementary Figure 8c), as their presence is entirely dependent on the adopted 3D fold and the observation of even a few of them is very restrictive in terms of available conformations of the system.

Thus, starting from the Zn^{2+}-titrated sample, the presence of 22 among 35 long-range NOEs from the original conditions was unambiguously confirmed (Table S2). Among the remaining 13 NOEs six were connectivities involving the G29-H1 proton, which we did not manage to observe in any of the four additional new samples, which was expected as this proton was already very broad in the original conditions – Supplementary Figure 7a. The remaining ones we considered uncertain either due to

signal overlap or because of a difficult to confirm resonance assignment. Importantly, no new sequential or long-range NOE was observed that was clearly absent in the original conditions. While already the observed NOEs do not leave much space for the structure in the presence of only Zn^{2+} to deviate significantly from the one observed in the original conditions, the comparison of the chemical shifts (CS) only reinforces this conclusion. Among 196 non-exchangeable protons assigned in both conditions (Table S3) only for one the chemical shift difference exceeds 0.1 ppm (A13-H2' with ΔCS= 0.141 ppm). This single atom is located far from the catalytic domain – in the GAA triloop capping Helix 2 – and thus the ΔCS is likely related to the difference in ionic strength between the two conditions (perhaps weak Na^{+} interaction with the GAA loop?).

For the Na^{+}-stabilized fold we were able to unambiguously observe 20 out of 35 long-range NOEs (Table S2). Moreover, also in this case the chemical shift comparison yielded very good agreement with the original conditions, however this time with 8 out of 196 compared atoms experiencing a ΔCS > 0.1 ppm. Out of these atoms three were located within the catalytic domain (C19-H1', T27-H1' and T27-H2') and five in Helix 2 (G7-H8, G7-H5', G8-H8, T9-Me and T17-H2''). Later Zn^{2+} binding geometry studies (see main text) revealed the G7-T17 base pair to be the likely site of the interaction which may explain why the CS in their vicinity turned out to be the most sensitive to the lack of a Zn^{2+} ion in the current sample.

The Mg^{2+}-stabilized sample produced the lowest quality 2D spectra and only 9 out of 35 originally observed long-range NOEs could be unambiguously confirmed. Also in terms of chemical shifts the largest differences with respect to the original conditions were observed, with 16 out of 185 ΔCS exceeding 0.1 ppm (five in the catalytic domain, five on residues forming the G-T base pair of Helix 2, four in the rest of Helix 2 and two in Helix 1). However, as many of these differences were located outside of the catalytic domain, some of them may reflect the differences in ionic strength between the sample. Indeed, when the ΔCS are instead calculated between the samples containing Mg^{2+} and Zn^{2+} (both without added NaCl) the agreement improves with 11 out of 177 values exceeding 0.1 ppm (five in the catalytic domain, six on residues forming the G-T base pair of Helix 2 and none in the rest of Helices 1 and 2). Thus, as was the case for Na^{+}, most chemical shift differences are measured around the G7-T17 base that we show in the text to host the metal binding site. They may thus reflect local structural differences dependent on the type of metal bound. The highest measured ΔCS belonged to T17-H2' which resonated at 0.934 ppm when Mg^{2+} was present and at 0.615 ppm in the samples containing Zn^{2+}. Despite these shifts being different they are actually both significantly upfield from the BMRB average (see previous section) and thus appear to tell a similar story of shielding by G18 aromatic ring, yet likely with two slightly different geometries.

Finally, for the fold stabilized by Pb^{2+} we managed to observe 14 out of 35 long range NOEs listed in Table 2. The chemical shift agreement with the sample stabilized by Zn^{2+} was exceptionally good with only three ΔCS > 0.1 ppm (two within the catalytic domain: C19-H1' and G25-H1') among 187 compared atoms. The agreement with the original conditions was also quite good with however with 10 out of 197 atoms experiencing a ΔCS > 0.1 ppm. Only four of the affected atoms were located within the catalytic domain (C19-H1', C19-H5, G25-H1' and T27-H2'), while the highest ΔCS – the only two over 0.2 ppm – were observed for the A13 residue of the GAA loop, likely related to the ionic strength difference between the samples as discussed above.

Overall, all four additional samples for which 2D NMR analysis was performed appear to feature very similar 8-17 DNAzyme folds, based on chemical shift agreements and the recurring observation of the same long-range NOEs (Supplementary Figure 8c).

Supplementary Note 4. Analysis of CD-titrations of phosphorothioate (PS) modified constructs

Most of the CD-titrations presented in this work were fitted using a simple 1:1 interaction model. However, the PS-modified variants of 8-17_short were synthesized as a mixture of two stereoisomers R_p and S_p, with potentially different affinities towards the studied metal ion, if one of the non-bridging oxygen atoms of the modified phosphate group is indeed involved in metal binding. In such cases the standard fitting procedure would yield only one "average" K_d value for the two and likely an unsatisfactory reproduction of the experimental titration curve. Thus, for the PS modified variants the titration data was fitted twice: once with the standard procedure, and for a second time with a modified model in which the affinity $K_{d,1}$ of one diastereoisomer (50% population) is assumed to be equal to that of the original 8-17_short (~15 µM) and the affinity of the other $K_{d,2}$ is fitted. If the two methods produce a similar fit quality and K_d values around that of 8-17_short, then the CD data point towards the phosphate group not being involved in the interaction.

In the case of a variant bearing the PS modification on the scissile phosphate (dubbed PS7 in Fig S14) the standard fitting yields a K_d value of 15 µM with a reasonable reproduction of the experimental profile. The application of the abovementioned alternative fitting procedure does not noticeably improve the fitting quality (nearly identical target function value) and the fitted $K_{d,2}$ is also 15 µM. Thus, the Zn^{2+} binding appears to be unaffected by the PS modification at the scissile phosphate.

In case of the second analyzed variant – with PS modification at T27 – the fitting with a single curve yielded a K_d value of 31 µM, while the fitting procedure allowing for two different K_d values gave $K_{d,1}$ equal to 63 µM and $K_{d,2}$ of 15 µM, confirming that the Zn^{2+} binding was affected by this modification.

Supplementary Discussion 1. The structure forming at higher Pb^{2+}-to-DNA molar ratios during 8-17_short titrations with Pb^{2+}

While a structural transition similar to that induced by other metal ions was observed in the initial steps of Pb^{2+} titrations of 8-17_short (Figure 7 and the SI section above), a second structural transition occurring only for this ion, was also observed by CD spectroscopy and by NMR at low DNAzyme concentrations starting at around 2 eq of Pb^{2+} and saturating at around 10 eq. The newly formed state exhibited a CD band centered at around 320 nm (Figure 7a), a feature very rare among known DNA structures.[5] The UV spectrum of the sample, measured alongside the CD signal (Supplementary Figure 9), also started exhibiting pronounced changes at around the same point in the titration (Supplementary Figure 9a; Pb^{2+} panel) with the extinction coefficient in the 220-250 nm spectral region increasing by over an order of magnitude and a new absorption band arising above 300 nm where light absorption by DNA is usually marginal. While some minor changes in the shape of the UV spectrum can occur during structural transitions in DNA the changes of this magnitude are extremely rare. Also, this effect was only observed for Pb^{2+} ions.

The newly forming structure also gave rise to a new set of NMR imino proton resonances, all resonating in the 11.5-12.5 ppm range (Figure 7c), completely distinct from the ones present in the earlier stages of the titration. The disappearance of all imino proton resonances originally present in the 12.5-14.0 ppm region, that are characteristic to standard Watson-Crick base pairs, signifies that the formation of the new state was accompanied by breaking of all Watson-Crick pairs within the molecule, even those present in the DNAzyme's two arms, and thus the destruction of all standard helical elements. The propensity of Pb^{2+} ions to promote DNA structures competitive to standard Watson-Crick paired

helices is well-known in the literature[6] and is attributed to direct Pb^{2+} coordination to electronegative atoms within DNA nucleobases which is accompanied by the disruption of base-pairing interactions. The formation of such metal-nucleobase complexes might be the source of the extensive changes observed in the UV spectrum. Given 8-17_short's particularly short helices, its secondary structure might be especially vulnerable to similar attacks by Pb^{2+} to which we attribute the gradual formation of this structure with just a few equivalents of Pb^{2+} being present. However, can any additional information be inferred from the available data about the exact nature of the conformation formed? While deeper investigations would be necessary, we would like to bring forward the idea that the product of the second structural transition might be a Pb^{2+}-stabilized G-quadruplex. While Pb^{2+} ions are not often cited as G-quadruplex inducing metal ions, their affinity to the cores of certain G-quadruplex structures is greater than even that of K^+ ions.[7] The classic G-quadruplex structure formed by the thrombin binding aptamer (TBA; 5'-GGTTGGTGTGGTTGG-3') was the first to be shown to bind Pb^{2+} preferentially to K^+ ions[7] and later structural investigations revealed that the two ions induced the same G-quadruplex topologies.[8] Interestingly, the Pb^{2+}-stabilized TBA G-quadruplex exhibited a CD band centered at 312 nm, similar to the structure we observe, as well as a set of NMR imino proton resonances at 11.9-12.3 ppm.[7] To the best of our knowledge the UV spectrum of Pb^{2+}-stabilized TBA G-quadruplex has not been published before and thus we have ourselves performed a CD and UV monitored titration of TBA with Pb^{2+} (Supplementary Figure 10). Interestingly, the UV spectral changes observed were qualitatively similar to the ones observed for 8-17_short. The similarities observed using three independent techniques (NMR, CD and UV spectroscopies) between Pb^{2+}-stabilized TBA G-quadruplex and the unknown structure formed by 8-17_short suggest that the latter could also be a Pb^{2+}-stabilized G-quadruplex.

Importantly, while for longer variants of 8-17 – specifically the ones form the crystallographic study[3] and the 2009 CD investigation[1] – we observe neither the CD band above 300 nm nor the appearance of a new set of imino protons (Figures 8, S11 and S12), changes in their UV spectra upon Pb^{2+} titration are once again present (Figures S9 and S11). They become apparent at higher Pb^{2+}-to-DNA molar ratios than for 8-17_short (at above around 10 eq of Pb^{2+}), but once again correspond to a significant increase in molar extinction coefficient in the 220-250 nm spectral region. This observation could suggest that, while not so prevalent as in case of 8-17_short, some binding of excess Pb^{2+} to nucleobases (and disruption of helical elements?) is also occurring for standard length constructs of the 8-17 DNAzyme when many-fold excess of Pb^{2+} is present.

Supplementary Discussion 2. Previous FRET and CD spectroscopy results in the light the results reported in this work

The new model for the interaction of 8-17 DNAzyme with divalent metal ions presented in Figure 9 is compatible with the literature FRET[9,10] and CD[1] observations, just as well as the classic two-structures explanation (Figure 1d).

The original FRET study has reported the shortening of the distance between the ends of the two substrate-binding arms in response to Zn^{2+} and Mg^{2+} but not to Pb^{2+}.[9] Similarly, the follow-up single-molecule FRET report observed a transient shortening of the said distance prior to catalytic cleavage in the presence of Zn^{2+} and Mg^{2+}, but once again not Pb^{2+}.[10] These results were classically seen as an indicator that ions such as Zn^{2+} or Mg^{2+} use a different (more compact) active state as compared to Pb^{2+}. If, however, we allow for the apo state of the enzyme be an ensemble of different conformations - on average more open than the compact catalytically-proficient state -, then the available bulk FRET data[9] can be seen as reflecting the population shift towards this state induced by Zn^{2+} or Mg^{2+}, but do

not exclude the possibility of this state being originally present to some extent for Pb^{2+} to use. The presence of a fraction of the folded state before divalent metal ions titrations is actually very likely, given that the buffer used in that FRET study (50 mM Na-HEPES acetate buffer)[9] contained Na^{+} ions, which at that time were not yet known to influence 8-17 DNAzyme folding.

For a similar interpretation to also fit with the single molecule FRET observations[10] one additional condition would need to be met i. e. the timescale of exchange between the different conformations forming the apo state ensemble would need to be significantly faster than the sampling rate of the experiment (100 ms). We believe such an assumption to be reasonable based on the following reasoning. The buffer used in the study contained 50 mM NaCl and thus with our current knowledge of Na^{+}-induced folding we can once again conclude that it contained a fraction of the folded state in exchange with other more open conformation(s). However, as the DNAzyme molecules before the addition of divalent metal ions displayed little variation in their FRET time traces (as seen e. g. in Figure 2a of [10]) the differences in FRET intensities between these states had to be averaged out by conformational exchange occurring multiple times between each measured time point. Within such an interpretation framework the addition of Zn^{2+} or Mg^{2+} would once again shift the equilibrium within the ensemble towards the folded state. This thanks to the postulated fast averaging would translate to each molecule spending on average more time in the folded conformation between the consecutive time points measured (ergodicity), leading to the observed increase of the smFRET signal. In case of moderate concentrations of Pb^{2+} (20 µM as used in the study) no such shift would occur according as Pb^{2+} would mostly bind to already preformed compact conformation (Figure 9). It has to be noted that the authors of the single molecule FRET study[10] briefly considered a similar interpretation in their discussion, yet decided to argue against it based on the multiple orders of magnitude wide mismatch between the exchange rates it would require (multiple exchange events occurring within 100 ms windows) and the typical times needed for the reaction to occur after the addition of Pb^{2+} (> 10 s). However, as the DNAzyme activation is a process involving more steps than just DNAzyme folding and metal binding, we find a similar mismatch between the timescales of folding/metal binding and those of enzymatic cleavage not to be improbable, if a different chemical event (e.g. the deprotonation of the **G14** nuclephile) constitutes the rate limiting step of the enzymatic cleavage mechanism.

One additional factor to consider when discussing the lack of Pb^{2+}-induced folding in the FRET experiments is that these studies employed very high Pb^{2+}-to-DNA molar ratios. In the bulk FRET study the DNAzyme concentration was <100 nM with Pb^{2+} concentration reaching up to 100 µM, yielding over thousandfold molar excess. In the smFRET the exact DNAzyme concentration was not reported, yet it was certainly submicromolar (the smFRET samples were diluted from 1 µM stocks) with 20 µM Pb^{2+}, yielding at least a twentyfold excess. At such high molar ratios Pb^{2+} might actually interfere with proper DNAzyme folding, as discussed above in the "*The structure forming at higher Pb^{2+}-to-DNA molar ratios during 8-17_short titrations with Pb^{2+}*" section.

Regarding CD spectroscopy the previous study[1] proposed Z-DNA formation in the compact Zn^{2+}-induced structure which we surprisingly did not observe in our solution structure. In that work the Z-DNA formation was inferred from the appearance of a weak negative CD band at > 290 nm. While negative CD ellipticity in this region is indeed observed for Z-DNA, it can also arise from other non-standard base stacking arrangements, including tandem GA mismatches in otherwise B-DNA duplexes.[11,12] While our solution structure of 8-17 DNAzyme does not feature Z-DNA, it does contain a tandem GA mismatch (A21-G29 and G6-A30), which might provide an explanation for the negative CD band. Further studies are however needed to settle this matter.

A more profound difference between our CD results and those reported previously[1] arises regarding the CD-monitored titrations using Pb^{2+} ions. While the original study did not observe significant

spectral changes upon Pb^{2+} titration, we on the other hand do for both the 8-17_short construct (Figure 7) and a full-length, bimolecular variant used previously for X-ray crystallography (Figure 8). In the case of the latter, the observed spectral changes are qualitatively extremely similar to those induced by other three metal ions tested here (Figure 8). To understand the source of this discrepancy we repeated the Pb^{2+} and Zn^{2+} titrations on the exact same 8-17 variant as used in the original study[1] and obtained the same results as the previous investigators did (Supplementary Figure 11). This means that the discrepancy between the two studies cannot be attributed to differences in experimental conditions, but rather the different CD properties of the various 8-17 DNAzyme constructs. Does this, however mean that some 8-17 DNAzyme variants undergo Pb^{2+}-induced folding while others do not? Such a possibility certainly cannot be discarded and the issue will require further study. On the other hand, one should also notice that even the CD spectral changes induced by the other metals (Na^{+}, Mg^{2+}, Zn^{2+}) are significantly more pronounced for the variants studied here (Figure 8) than they were for the variant used in the original study (Figure 6 in [1]). It cannot thus be excluded that the Pb^{2+}-induced folding of that variant simply produced CD spectral changes that are too subtle to measure accurately.

Supplementary References

1. Mazumdar, D. *et al.* Activity, Folding and Z-DNA Formation of the 8-17 DNAzyme in the Presence of Monovalent Ions. *J. Am. Chem. Soc.* **131**, 5506–5515 (2009).
2. Zuker, M. Mfold web server for nucleic acid folding and hybridization prediction. *Nucleic Acids Res.* **31**, 3406–3415 (2003).
3. Liu, H. *et al.* Crystal structure of an RNA-cleaving DNAzyme. *Nat. Commun.* **8**, 2006 (2017).
4. Yoshizawa, S., Kawai, G., Watanabe, K., Miura, K. & Ichiro, H. GNA Trinucleotide Loop Sequences Producing Extraordinarily Stable DNA Minihairpins. *Biochemistry* **36**, 4761–4767 (1997).
5. Vorlíčková, M. *et al.* Circular Dichroism Spectroscopy of DNA: From Duplexes to Quadruplexes. *Chirality* **24**, 691–698 (2012).
6. Billet, B. *et al.* Aptamer Switches Regulated by Post-Transition/Transition Metal Ions. *Angew. Chemie Int. Ed.* **60**, 12346–12350 (2021).
7. Smirnov, I. & Shafer, R. H. Lead is unusually effective in sequence-specific folding of DNA. *J. Mol. Biol.* **296**, 1–5 (2000).
8. Liu, H. *et al.* Structure-guided development of Pb2+-binding DNA aptamers. *Sci. Reports 2022 121* **12**, 1–11 (2022).
9. Kim, H.-K. *et al.* Metal-Dependent Global Folding and Activity of the 8-17 DNAzyme Studied by Fluorescence Resonance Energy Transfer. *J. Am. Chem. Soc.* **129**, 6896–6902 (2007).
10. Kim, H. K., Rasnik, I., Liu, J., Ha, T. & Lu, Y. Dissecting metal ion–dependent folding and catalysis of a single DNAzyme. *Nat. Chem. Biol. 2007 312* **3**, 763–768 (2007).
11. Lane, A., Martin, S. R., Ebel, S. & Brown, T. Solution conformation of a deoxynucleotide containing tandem G.A mismatched base pairs and 3'-overhanging ends in d(GTGAACTT)2. *Biochemistry* **32**, 3829 (1993).
12. Lane, A., Ebel, S. & Brown, T. Properties of multiple G.A mismatches in stable oligonucleotide duplexes. *Eur. J. Biochem.* **220**, 717–727 (1994).
