# Supplementary material for: The 8-17 DNAzyme can operate in a single active structure regardless of metal ion cofactor
Source: Nat Commun. 2024 May 17;15:4218. doi: 10.1038/s41467-024-48638-x (PMC11101458; doi:10.1038/s41467-024-48638-x)
Supplement: Supplementary file 3 — Description of Additional Supplementary Files [file 41467_2024_48638_MOESM3_ESM.pdf]

Description of Additional Supplementary Files

File Name: Supplementary Data 1
Description: NMR chemical shifts of non-exchangeable ^{1}H and ^{31}P nuclei.
Non-exchangeable ^{1}H and ^{31}P chemical shifts measured for 8-17_short in sodium cacodylate buffer (pH 6.0) in the presence of different concentrations of divalent and monovalent ions.

File Name: Supplementary Data 2
Description: NMR chemical shifts of water exchangeable ^{1}H nuclei.
Exchangeable ^{1}H chemical shifts measured for 8-17_short in sodium cacodylate buffer (pH 6.0) in the presence of different concentrations of divalent and monovalent ions.
