# Supplementary material for: The 8-17 DNAzyme can operate in a single active structure regardless of metal ion cofactor
Source: Nat Commun. 2024 May 17;15:4218. doi: 10.1038/s41467-024-48638-x (PMC11101458; doi:10.1038/s41467-024-48638-x)
Supplement: Supplementary file 4 — Supplementary Data 1 [file 41467_2024_48638_MOESM4_ESM.pdf]

Supplementary Data 1. Non-exchangeable ^{1}H and ^{31}P chemical shifts measured for 8-17_short in sodium cacodylate buffer (pH 6.0) in the presence of different concentrations of divalent and monovalent ions.[a,b]

		Sample conditions									
Metal ions present:		4 mM Zn^{2+}, 200 mM Na^{+}	400 mM Na^{+}	1.5 mM Zn^{2+}	7.5 mM Mg^{2+}	3.5 mM Pb^{2+}	400 mM Na^{+}, 1.5 mM Zn^{2+}	400 mM Na^{+}, 3.0 mM Zn^{2+}	400 mM Na^{+}, 4.5 mM Zn^{2+}	3.0 mM Zn^{2+}	4.5 mM Zn^{2+}
DNA concentration:		2.2 mM	1.5 mM	1.5 mM	1.5 mM	1.5 mM	1.5 mM	1.5 mM	1.5 mM	1.5 mM	1.5 mM
Temperature:		35 °C	20 °C	15 °C	15 °C	15 °C	20 °C	20 °C	20 °C	15 °C	15 °C
Comment:		conditions used for structure determination	DNAzyme folding with Na^{+} only	DNAzyme folding with Zn^{+} only	DNAzyme folding with Na^{+} only	DNAzyme folding with Zn^{+} only	titration of the Na^{+}-stabilized DNAzyme with Zn^{2+}			titration of the Zn^{2+}-stabilized DNAzyme with additional equivalents of the ion	
Residue	Atom										
C1	H1'	5.817	5.762	5.794	5.788	5.796	5.764	5.754	5.756	5.793	5.788
C1	H2'	1.991	1.987	2.023	2.016	2.031	1.991	1.982	1.979	2.026	2.027
C1	H2''	2.454	2.438	2.465	2.464	2.469	2.442	2.436	2.434	2.468	2.468
C1	H3'	4.736	4.726	4.746	4.746	4.745	4.727	4.722	4.723	4.745	4.745
C1	H4'	4.105	4.088	---	---	---	4.09	---	---	---	---
C1	H5'	3.776	---	---	---	---	---	---	---	---	---
C1	H5''	3.75	---	---	---	---	---	---	---	---	---
C1	P	---	---	---	---	---	---	---	---	---	---
C1	H5	5.962	5.925	5.963	5.96	5.968	5.929	5.918	5.918	5.965	5.961
C1	H6	7.684	7.654	7.679	7.683	7.698	7.659	7.655	7.656	7.684	7.681
G2	H1'	5.95	5.92	5.932	5.925	5.943	5.92	5.914	5.913	5.93	5.927
G2	H2'	2.734	2.728	2.737	2.739	2.731	2.729	2.729	2.732	2.74	2.74
G2	H2''	2.773	2.745	2.759	2.739	2.765	2.749	2.744	2.741	2.761	2.761
G2	H3'	5.019	5.007	5.015	5.015	5.017	5.011	5.011	5.011	5.018	5.018
G2	H4'	4.401	4.39	---	---	---	4.39	---	---	---	---
G2	H5'	---	---	---	---	---	---	---	---	---	---
G2	H5''	4.038	---	---	---	---	---	---	---	---	---

G2	P	-4.201	-4.064	-3.99	-4.065	-4.061	-4.077	-4.066	-4.066	-4.053	-4.034
G2	H8	8.024	8.01	8.024	8.026	8.029	8.013	8.013	8.017	8.031	8.032
C3	H1'	6.063	6.033	6.054	6.058	6.047	6.035	6.036	6.041	6.063	6.065
C3	H2'	2.15	2.136	2.145	2.147	2.136	2.143	2.145	2.155	2.157	2.163
C3	H2''	2.495	2.468	2.496	2.492	2.484	2.469	2.467	2.461	2.503	2.505
C3	H3'	4.892	4.877	4.894	4.9	4.889	4.886	4.888	4.891	4.898	4.902
C3	H4'	4.288	4.282	---	---	---	4.288	---	---	---	---
C3	H5'	4.241	---	---	---	---	---	---	---	---	---
C3	H5''	4.171	---	---	---	---	---	---	---	---	---
C3	P	-4.338	-4.176	-4.074	-4.153	-4.124	-4.188	-4.182	-4.182	-4.147	-4.111
C3	H5	5.463	5.456	5.456	5.463	5.459	5.463	5.463	5.463	5.458	5.459
C3	H6	7.493	7.468	7.478	7.486	7.477	7.479	7.482	7.487	7.488	7.491
C4	H1'	5.626	5.584	5.604	5.557	5.583	5.6	5.598	5.598	5.607	5.608
C4	H2'	1.762	1.806	1.808	1.881	1.835	1.787	1.769	1.748	1.785	1.769
C4	H2''	2.184	2.193	2.205	2.248	2.214	2.185	2.171	2.16	2.192	2.183
C4	H3'	4.82	4.813	4.824	4.84	4.826	4.815	4.813	4.809	4.824	4.822
C4	H4'	4.091	4.09	---	---	---	4.093	---	---	---	---
C4	H5'	---	---	---	---	---	---	---	---	---	---
C4	H5''	---	---	---	---	---	---	---	---	---	---
C4	P	-4.261	-4.079	---	---	---	-4.098	-4.111	-4.111	---	---
C4	H5	5.624	5.619	5.616	5.629	5.619	5.612	5.609	5.608	5.617	5.617
C4	H6	7.354	7.355	7.359	7.398	7.376	7.34	7.335	7.333	7.358	7.354
G5	H1'	5.96	5.937	5.949	5.953	5.952	5.956	5.958	5.96	5.958	5.961
G5	H2'	2.18	2.181	2.192	---	---	2.157	2.162	2.171	2.211	2.21
G5	H2''	2.426	2.431	2.438	---	---	2.428	2.423	2.42	2.43	2.437
G5	H3'	4.974	4.965	4.974	4.995	4.981	4.966	4.971	4.97	4.977	4.977
G5	H4'	4.387	---	---	---	---	---	---	---	---	---
G5	H5'	---	---	---	---	---	---	---	---	---	---
G5	H5''	---	---	---	---	---	---	---	---	---	---
G5	P	-4.249	-4.039	---	---	---	-4.076	-4.086	-4.097	---	---

G5	H8	7.455	7.474	---	---	---	7.432	7.427	7.428	---	---
G6	H1'	5.731	5.764	5.758	5.851	5.811	5.712	5.704	5.709	5.766	5.775
G6	H2'	3.134	3.084	3.105	3.048	3.037	3.128	3.131	3.131	3.121	3.118
G6	H2''	3.148	3.152	3.184	3.244	3.209	3.136	3.132	3.132	3.188	3.164
G6	H3'	5.267	5.218	5.258	5.229	5.242	5.256	5.262	5.265	5.266	5.268
G6	H4'	4.962	---	---	---	---	---	---	---	---	---
G6	H5'	---	---	---	---	---	---	---	---	---	---
G6	H5''	---	---	---	---	---	---	---	---	---	---
G6	P	-4.978	---	---	---	---	-4.794	-4.822	-4.841	---	---
G6	H8	8.303	8.273	8.313	8.311	8.324	8.295	8.307	8.319	8.34	8.343
G7	H1'	6.394	6.339	6.394	6.268	6.342	6.398	6.399	6.393	6.394	6.385
G7	H2'	3.251	3.223	3.226	3.11	3.178	3.241	3.24	3.236	3.229	3.228
G7	H2''	3.027	2.966	3.014	3.002	3.017	3.008	3.022	3.028	3.029	3.026
G7	H3'	4.922	4.885	4.898	4.982	4.954	4.887	4.897	4.904	4.898	4.904
G7	H4'	4.524	4.494	4.527	---	---	---	---	---	---	---
G7	H5'	4.659	4.529	---	---	---	---	---	---	---	---
G7	H5''	4.387	4.342	---	---	---	---	---	---	---	---
G7	P	-3.983	-3.809	-3.813	---	-3.711	-3.901	-3.891	-3.877	-3.879	-3.846
G7	H8	8.481	8.212	8.46	8.254	8.393	8.444	8.477	8.484	8.479	8.474
G8	H1'	6.025	6.041	6.012	6.037	6.01	6.009	6.002	6.001	6.012	6.01
G8	H2'	2.581	2.532	2.565	2.572	2.547	2.565	2.57	2.573	2.571	2.574
G8	H2''	2.835	2.807	2.838	2.819	2.811	2.832	2.835	2.834	2.842	2.841
G8	H3'	4.915	4.876	4.89	4.905	4.87	4.895	4.9	4.902	4.895	4.9
G8	H4'	4.365	---	---	---	---	---	---	---	---	---
G8	H5'	---	---	---	---	---	---	---	---	---	---
G8	H5''	---	---	---	---	---	---	---	---	---	---
G8	P	-4.283	-3.872	---	---	---	-4.101	-4.135	-4.165	---	---
G8	H8	7.801	7.616	7.76	7.701	7.627	7.77	7.798	7.808	7.779	7.785
T9	H1'	6.074	6.1	6.069	6.081	6.105	6.062	6.053	6.051	6.065	6.064
T9	H2'	1.876	1.888	1.864	1.882	1.914	1.851	1.84	1.84	1.864	1.861

T9	H2''	2.44	2.435	2.423	2.414	2.444	2.424	2.419	2.419	2.424	2.422
T9	H3'	4.792	4.794	4.786	4.803	4.807	4.779	4.772	4.768	4.783	4.78
T9	H4'	4.216	---	---	---	---	---	---	---	---	---
T9	H5'	---	---	---	---	---	---	---	---	---	---
T9	H5''	---	---	---	---	---	---	---	---	---	---
T9	P	-4.481	-4.493	---	---	---	-4.343	-4.308	-4.303	---	---
T9	Me	1.413	1.297	1.375	1.335	1.29	1.394	1.409	1.413	1.386	1.386
T9	H6	7.457	7.462	7.438	7.398	7.484	7.437	7.434	7.434	7.442	7.442
C10	H1'	6.036	6.018	6.015	6.008	6.017	6.015	6.015	6.017	6.019	6.018
C10	H2'	1.711	1.737	1.758	1.79	1.76	1.741	1.737	1.742	1.758	1.772
C10	H2''	2.259	2.242	2.281	2.252	2.254	2.248	2.248	2.248	2.28	2.28
C10	H3'	4.827	4.84	4.836	4.844	4.839	---	---	---	4.836	4.836
C10	H4'	4.245	---	---	---	---	---	---	---	---	---
C10	H5'	---	---	---	---	---	---	---	---	---	---
C10	H5''	---	---	---	---	---	---	---	---	---	---
C10	P	-4.643	-4.463	---	---	---	-4.522	-4.529	-4.532	---	---
C10	H5	5.48	5.445	5.441	5.416	5.44	5.454	5.455	5.457	5.445	5.442
C10	H6	7.232	7.218	7.234	7.231	7.225	7.222	7.222	7.221	7.235	7.233
G11	H1'	5.36	5.337	5.345	5.341	5.342	5.339	5.336	5.334	5.346	5.344
G11	H2'	2.702	2.703	2.704	2.715	2.708	2.697	2.705	2.705	2.704	2.704
G11	H2''	2.563	2.558	2.566	2.572	2.566	2.558	2.555	2.554	2.574	2.574
G11	H3'	4.92	4.917	4.927	---	4.927	4.94	4.944	4.944	4.927	4.926
G11	H4'	4.486	---	---	---	---	---	---	---	---	---
G11	H5'	---	---	---	---	---	---	---	---	---	---
G11	H5''	---	---	---	---	---	---	---	---	---	---
G11	P	-4.974	---	---	---	---	---	---	---	---	---
G11	H8	8.115	8.122	8.138	8.146	8.139	8.125	8.128	8.134	8.147	8.148
A12	H1'	5.991	5.975	5.974	5.972	5.969	5.978	5.979	5.979	5.976	5.973
A12	H2'	2.293	2.263	2.256	2.249	2.253	2.261	2.26	2.261	2.256	2.254
A12	H2''	2.345	2.317	2.324	2.328	2.326	2.322	2.321	2.321	2.326	2.321

A12	H3'	4.595	4.574	4.58	4.575	4.577	4.578	4.577	4.577	4.58	4.578
A12	H4'	2.147	---	---	2.057	---	---	---	---	---	---
A12	H5'	3.396	3.375	3.392	3.403	3.401	---	---	---	3.4	3.402
A12	H5''	3.087	3.069	3.086	3.101	3.095	---	---	---	3.091	3.084
A12	P	-4.659	---	---	---	---	---	---	---	---	---
A12	H8	8.15	8.127	8.136	8.139	8.137	8.128	8.126	8.127	8.138	8.133
A12	H2	---	---	---	---	---	---	---	---	---	---
A13	H1'	6.24	6.233	6.273	6.28	6.285	6.242	6.239	6.239	6.271	6.271
A13	H2'	2.863	2.834	2.722	2.729	2.726	2.84	2.838	2.838	2.728	2.73
A13	H2''	2.862	2.834	2.855	2.859	2.866	2.84	2.838	2.838	2.851	2.852
A13	H3'	4.86	4.839	---	5.013	5.085	---	---	---	---	---
A13	H4'	4.366	---	---	---	---	---	---	---	---	---
A13	H5'	---	---	---	---	---	---	---	---	---	---
A13	H5''	---	---	---	---	---	---	---	---	---	---
A13	P	-4.756	-4.641	---	---	---	-4.655	-4.648	-4.648	---	---
A13	H8	7.986	7.976	---	8.276	8.268	7.976	7.982	7.981	---	---
A13	H2	8.088	8.06	---	---	8.069	---	---	---	---	---
G14	H1'	5.077	5.007	5.026	4.992	5.034	5.018	5.02	5.024	5.03	5.025
G14	H2'	2.637	2.635	2.651	2.648	2.643	2.659	2.66	2.661	2.656	2.659
G14	H2''	2.579	2.537	2.546	2.542	2.543	2.55	2.553	2.554	2.551	2.549
G14	H3'	4.913	4.901	4.903	4.912	4.902	4.913	4.914	4.914	4.905	4.908
G14	H4'	4.367	---	---	---	---	---	---	---	---	---
G14	H5'	4.274	---	---	---	---	---	---	---	---	---
G14	H5''	4.136	---	---	---	---	---	---	---	---	---
G14	P	-5.189	---	---	---	---	---	---	---	---	---
G14	H8	8.06	8.054	8.081	8.071	8.07	8.078	8.082	8.085	8.093	8.096
A15	H1'	6.249	6.263	6.253	6.264	6.269	6.247	6.242	6.241	6.251	6.25
A15	H2'	2.588	2.581	2.581	2.615	2.583	2.579	2.579	2.579	2.581	2.581
A15	H2''	2.775	2.797	2.777	2.801	2.812	2.766	2.758	2.758	2.774	2.773
A15	H3'	5.034	5.029	5.038	5.041	5.031	5.034	5.034	5.033	5.038	5.039

A15	H4'	4.468	---	---	---	---	---	---	---	---	---
A15	H5'	4.195	---	---	---	---	---	---	---	---	---
A15	H5''	4.121	---	---	---	---	---	---	---	---	---
A15	P	-3.699	-3.48	-3.416	-3.509	-3.502	-3.493	-3.479	-3.486	-3.488	-3.455
A15	H8	8.193	8.177	---	8.206	8.182	8.196	8.198	8.198	---	---
A15	H2	8.071	8.055	8.055	8.049	8.045	8.062	8.061	8.06	8.055	8.054
C16	H1'	5.95	5.885	5.93	5.919	5.882	5.933	5.937	5.937	5.933	5.933
C16	H2'	1.975	1.94	1.974	1.913	1.924	1.982	1.98	1.978	1.976	1.976
C16	H2''	2.334	2.31	2.317	2.338	2.324	2.308	2.306	2.307	2.317	2.315
C16	H3'	4.813	4.801	4.814	4.795	4.785	4.816	4.816	4.814	4.814	4.814
C16	H4'	4.198	---	---	---	---	---	---	---	---	---
C16	H5'	---	---	---	---	---	---	---	---	---	---
C16	H5''	---	---	---	---	---	---	---	---	---	---
C16	P	-4.393	-4.296	---	---	---	---	---	---	---	---
C16	H5	5.022	5.037	5.001	5.025	5.02	5.005	4.998	4.996	4.996	4.995
C16	H6	7.231	7.213	7.245	7.212	7.217	7.247	7.248	7.245	7.246	7.248
T17	H1'	5.444	5.454	5.428	5.425	5.462	5.417	5.407	5.407	5.425	5.425
T17	H2'	0.615	0.536	0.615	0.934	0.652	0.552	0.562	0.575	0.632	0.632
T17	H2''	1.566	1.72	1.582	1.759	1.668	1.551	1.516	1.505	1.555	1.55
T17	H3'	4.399	4.39	4.397	4.498	4.42	4.374	4.373	4.373	4.397	4.397
T17	H4'	4.183	4.165	4.168	4.131	---	4.182	4.183	4.179	---	---
T17	H5'	4.136	4.116	4.122	4.109	4.121	4.123	4.121	4.118	---	---
T17	H5''	3.7	3.697	3.702	3.798	3.726	3.687	3.683	3.684	3.707	3.71
T17	P	-5.116	-4.889	-4.923	-4.88	-4.873	-5.006	-5.019	-5.019	-4.983	-4.955
T17	Me	1.343	1.319	1.34	1.373	1.306	1.325	1.322	1.32	1.335	1.333
T17	H6	6.969	6.953	6.974	7.035	7	6.956	6.956	6.957	6.975	6.976
G18	H1'	5.38	5.418	5.383	5.563	5.464	5.357	5.345	5.345	5.376	5.375
G18	H2'	2.853	2.812	2.841	2.782	2.826	2.84	2.849	2.852	2.85	2.849
G18	H2''	2.494	2.516	2.495	2.59	2.544	2.476	2.47	2.472	2.494	2.492
G18	H3'	4.98	4.957	4.973	4.973	4.965	4.971	4.973	4.975	4.973	4.973

G18	H4'	4.259	---	---	---	---	---	---	---	---	---
G18	H5'	---	---	---	---	---	---	---	---	---	---
G18	H5''	3.841	---	---	---	---	---	---	---	---	---
G18	P	-4.599	-4.394	---	---	---	-4.41	-4.408	-4.408	---	---
G18	H8	7.974	7.958	7.974	7.959	7.973	7.973	7.978	7.982	7.979	7.984
C19	H1'	6.251	6.088	6.205	---	6.057	6.23	6.247	6.247	6.216	6.216
C19	H2'	2.035	1.955	2.013	2.071	1.993	2.003	2.02	2.03	2.033	2.031
C19	H2''	2.405	2.305	2.35	2.315	2.343	2.334	2.354	2.375	2.381	2.383
C19	H3'	4.969	4.87	4.952	4.891	4.887	4.941	4.951	4.957	4.953	4.954
C19	H4'	4.443	4.383	4.413	---	---	---	---	---	---	---
C19	H5'	4.359	---	---	---	---	---	---	---	---	---
C19	H5''	---	---	---	---	---	---	---	---	---	---
C19	P	-2.958	-2.956	-2.808	---	---	-2.814	-2.799	-2.791	-2.904	-2.871
C19	H5	5.877	5.792	5.861	5.739	5.775	5.871	5.881	5.881	5.865	5.857
C19	H6	7.6	7.543	7.579	7.544	7.561	7.577	7.584	7.591	7.589	7.584
C20	H1'	6.469	6.411	6.438	6.56	6.467	6.411	6.413	6.42	6.447	6.444
C20	H2'	2.774	2.776	2.812	2.631	2.725	2.801	2.801	2.805	2.766	2.772
C20	H2''	2.775	2.758	2.772	2.814	2.778	2.771	2.771	2.767	2.766	2.772
C20	H3'	5.173	5.127	5.156	5.137	5.137	5.15	5.154	5.154	5.158	5.155
C20	H4'	4.822	---	---	---	---	---	---	---	---	---
C20	H5'	4.362	---	---	---	---	---	---	---	---	---
C20	H5''	4.087	4.075	4.065	---	4.092	---	---	---	---	---
C20	P	-4.979	---	-4.888	---	---	-4.915	-4.91	-4.887	-4.894	-4.832
C20	H5	5.886	5.839	5.873	5.864	5.85	5.862	5.862	5.86	5.87	5.855
C20	H6	7.859	7.832	7.874	7.816	7.832	7.852	7.852	7.845	7.865	7.849
A21	H1'	6.333	6.299	6.323	6.318	6.315	6.313	6.313	6.312	6.324	6.315
A21	H2'	2.171	2.126	2.134	---	2.09	2.17	2.176	2.175	2.138	2.127
A21	H2''	2.825	2.802	2.808	2.766	2.78	2.808	2.811	2.811	2.809	2.8
A21	H3'	5.213	5.194	5.2	5.196	5.186	5.208	5.21	5.209	5.199	5.198
A21	H4'	4.351	4.352	4.365	4.371	4.361	---	---	---	---	---

A21	H5'	4.476	4.437	4.469	4.423	4.443	---	---	---	---	---
A21	H5''	4.219	4.204	4.208	4.2	4.2	---	---	---	---	---
A21	P	-3.094	---	-2.949	---	---	-3.015	-2.987	-2.951	-3.109	-3.084
A21	H8	7.876	7.893	7.889	---	7.942	7.842	7.834	7.832	7.882	7.867
A21	H2	8.195	8.15	8.18	8.173	8.181	---	---	---	---	---
G22	H1'	5.96	5.931	5.965	5.964	5.95	5.951	5.951	5.947	5.964	5.96
G22	H2'	2.496	2.49	2.487	2.466	2.464	2.477	2.472	2.47	2.483	2.489
G22	H2''	2.525	2.516	2.537	2.548	2.54	2.536	2.54	2.537	2.539	2.538
G22	H3'	4.707	4.69	4.693	4.709	4.689	4.697	4.698	4.7	4.696	4.7
G22	H4'	4.372	---	---	---	---	---	---	---	---	---
G22	H5'	---	---	---	---	---	---	---	---	---	---
G22	H5''	---	---	---	---	---	---	---	---	---	---
G22	P	-4.146	---	-3.88	---	---	-3.867	-3.898	-3.922	-3.914	-3.869
G22	H8	8.005	7.967	8.006	7.954	7.994	7.976	7.972	7.967	8.001	7.987
C23	H1'	5.507	5.455	5.488	5.469	5.507	5.46	5.464	5.467	5.492	5.478
C23	H2'	1.83	1.782	1.813	1.813	1.825	1.783	1.779	1.783	1.814	1.801
C23	H2''	2.303	2.299	2.293	2.305	2.297	2.299	2.292	2.289	2.293	2.319
C23	H3'	4.49	4.46	4.468	4.48	4.476	4.455	4.455	4.455	4.467	4.462
C23	H4'	3.532	---	---	---	---	---	---	---	---	---
C23	H5'	3.79	---	---	---	---	---	---	---	---	---
C23	H5''	3.668	---	---	---	---	---	---	---	---	---
C23	P	-3.73	-3.525	-3.495	-3.592	-3.502	-3.55	-3.543	-3.54	-3.55	-3.514
C23	H5	6.244	6.154	6.223	6.199	6.226	6.166	6.175	6.179	6.225	6.187
C23	H6	7.683	7.584	7.65	7.649	7.656	7.597	7.603	7.605	7.65	7.62
G24	H1'	5.722	5.651	5.699	5.757	5.66	5.724	5.752	5.768	5.732	5.749
G24	H2'	2.577	2.593	2.563	2.657	2.633	2.555	2.567	2.584	2.582	2.591
G24	H2''	2.829	2.804	2.795	2.889	2.845	2.799	2.815	2.834	2.823	2.834
G24	H3'	4.731	4.731	4.746	4.753	4.74	4.72	4.717	4.722	4.748	4.748
G24	H4'	4.291	---	---	---	4.287	---	---	---	---	---
G24	H5'	3.85	---	---	---	3.851	---	---	---	---	---

G24	H5''	3.567	---	---	---	3.594	---	---	---	---	---
G24	P	-4.113	-3.83	-3.798	-3.867	-3.905	-3.856	-3.905	-3.954	-3.922	-3.911
G24	H8	7.73	7.676	7.68	7.735	7.735	7.663	7.705	7.751	7.722	7.731
G25	H1'	5.218	5.307	5.185	5.337	5.343	5.154	5.137	5.144	5.185	5.185
G25	H2'	2.459	2.447	2.481	2.403	2.461	2.462	2.45	2.441	2.466	2.462
G25	H2''	2.328	2.348	2.315	2.361	2.401	2.298	2.295	2.295	2.316	2.318
G25	H3'	4.794	4.798	4.786	4.812	4.815	4.78	4.776	4.774	4.785	4.784
G25	H4'	4.031	---	---	---	---	---	---	---	---	---
G25	H5'	---	---	---	---	---	---	---	---	---	---
G25	H5''	---	---	---	---	---	---	---	---	---	---
G25	P	---	---	---	---	---	---	---	---	---	---
G25	H8	7.736	7.738	7.758	7.733	7.764	7.734	7.734	7.736	7.76	7.759
C26	H1'	6.076	6.05	6.079	6.083	6.078	6.056	6.05	6.045	6.075	6.069
C26	H2'	2.21	2.276	2.219	2.272	2.201	2.208	2.196	2.183	2.214	2.209
C26	H2''	2.461	2.403	2.44	2.454	2.464	2.435	2.443	2.452	2.45	2.451
C26	H3'	4.72	4.737	4.727	4.758	4.715	4.711	4.704	4.701	4.725	4.722
C26	H4'	3.696	---	---	3.663	---	---	---	---	---	---
C26	H5'	3.809	---	---	---	---	---	---	---	---	---
C26	H5''	3.777	---	---	---	---	---	---	---	---	---
C26	P	-3.442	---	-3.188	---	---	-3.233	-3.202	-3.202	-3.308	-3.283
C26	H5	5.659	5.602	5.642	5.578	5.603	5.645	5.652	5.651	5.647	5.641
C26	H6	7.458	7.376	7.452	7.394	7.432	7.435	7.44	7.439	7.453	7.449
T27	H1'	6.131	6.022	6.123	6.046	6.047	6.131	6.141	6.14	6.127	6.126
T27	H2'	1.626	1.852	---	1.796	1.732	1.647	1.607	1.597	---	---
T27	H2''	2.306	2.318	---	2.309	2.308	2.288	2.279	2.279	---	---
T27	H3'	4.651	4.624	4.663	4.612	4.607	4.66	4.659	4.653	4.656	4.654
T27	H4'	4.197	---	---	---	---	---	---	---	---	---
T27	H5'	4.031	---	---	---	---	---	---	---	---	---
T27	H5''	---	---	---	---	---	---	---	---	---	---
T27	P	-4.902	-4.593	---	---	---	-4.845	-4.871	-4.868	---	---

T27	Me	1.652	1.643	1.647	1.659	1.657	1.645	1.644	1.645	1.647	---
T27	H6	7.244	7.264	7.239	7.285	7.296	7.231	7.227	7.227	7.239	7.239
C28	H1'	6.008	5.972	5.976	5.983	5.976	5.985	5.984	5.986	5.984	5.984
C28	H2'	2.146	2.11	2.158	---	2.134	2.158	2.164	2.164	2.165	2.163
C28	H2''	2.393	---	---	---	2.35	2.381	2.381	2.381	---	---
C28	H3'	4.878	4.865	4.881	4.871	---	4.883	4.882	4.882	4.883	4.884
C28	H4'	4.278	---	---	---	---	---	---	---	---	---
C28	H5'	3.964	---	---	---	---	---	---	---	---	---
C28	H5''	---	---	---	---	---	---	---	---	---	---
C28	P	-4.75	-4.635	---	---	---	-4.654	-4.64	-4.637	---	---
C28	H5	5.617	5.526	5.573	5.544	5.576	5.571	5.575	5.576	5.574	5.567
C28	H6	7.3	7.311	7.308	7.328	7.333	7.288	7.279	7.278	7.303	7.3
G29	H1'	---	---	---	---	---	---	---	---	---	---
G29	H2'	---	---	---	---	---	---	---	---	---	---
G29	H2''	---	---	---	---	---	---	---	---	---	---
G29	H3'	5.013	4.989	5	4.989	4.989	5.002	5.001	5.001	5	4.998
G29	H4'	4.509	---	---	---	---	---	---	---	---	---
G29	H5'	---	---	---	---	---	---	---	---	---	---
G29	H5''	---	---	---	---	---	---	---	---	---	---
G29	P	-5.175	---	---	---	---	---	---	---	---	---
G29	H8	8.111	8.112	8.135	---	8.142	8.123	8.126	8.127	8.147	8.15
A30	H1'	5.149	5.134	5.151	---	---	5.097	5.091	5.094	5.151	5.147
A30	H2'	1.424	1.446	1.413	---	---	1.326	1.326	---	1.427	1.416
A30	H2''	2.327	2.324	---	---	---	2.287	2.277	2.273	---	---
A30	H3'	4.675	4.655	4.668	---	4.693	4.643	4.641	4.641	4.666	4.662
A30	H4'	3.335	3.282	3.304	3.314	3.345	---	---	---	---	---
A30	H5'	3.944	3.931	3.94	3.943	3.951	---	---	---	---	---
A30	H5''	3.677	3.654	3.653	3.645	3.661	---	---	---	---	---
A30	P	-2.817	-2.594	-2.59	-2.686	-2.68	-2.622	-2.624	-2.628	-2.671	-2.638
A30	H8	8.122	8.073	8.111	---	8.145	8.051	8.045	8.038	8.098	8.065

A30	H2	8.476	8.379	8.446	8.393	8.415	---	---	---	---	---
C31	H1'	5.01	4.916	4.977	---	---	4.894	4.897	4.898	4.99	4.976
C31	H2'	2.114	2.164	2.157	2.125	2.134	2.186	2.171	2.137	2.13	2.119
C31	H2''	2.307	2.289	2.306	2.291	2.309	2.298	2.294	2.29	2.305	2.298
C31	H3'	4.799	4.787	4.801	---	4.801	4.79	4.786	4.782	4.795	4.791
C31	H4'	3.98	3.975	---	3.989	---	---	---	---	---	---
C31	H5'	---	---	---	---	---	---	---	---	---	---
C31	H5''	3.837	---	---	---	---	---	---	---	---	---
C31	P	-4.943	-4.733	---	---	---	-4.788	-4.78	-4.775	---	---
C31	H5	5.757	5.702	5.712	---	5.668	5.753	5.756	5.757	5.718	5.726
C31	H6	7.402	7.408	7.411	---	7.399	7.438	7.434	7.425	7.398	7.403
G32	H1'	5.717	5.672	5.677	5.657	5.717	5.69	5.702	5.715	5.697	5.701
G32	H2'	2.732	2.713	2.732	2.73	2.709	2.721	2.723	2.729	2.74	2.742
G32	H2''	2.838	2.804	2.816	2.81	2.82	2.82	2.827	2.835	2.832	2.836
G32	H3'	5.046	5.032	5.043	5.043	5.043	5.042	5.043	5.046	5.045	5.045
G32	H4'	4.426	---	---	---	---	---	---	---	---	---
G32	H5'	4.133	---	---	---	---	---	---	---	---	---
G32	H5''	4.033	---	---	---	---	---	---	---	---	---
G32	P	-3.736	-3.44	-3.438	---	---	-3.377	-3.406	-3.458	-3.57	-3.552
G32	H8	7.902	7.829	7.864	7.861	7.879	7.839	7.861	7.889	7.9	7.906
G33	H1'	5.906	5.886	5.916	5.921	5.872	5.882	5.876	5.875	5.916	5.914
G33	H2'	2.595	2.591	2.597	2.604	2.596	2.593	2.589	2.586	2.596	2.595
G33	H2''	2.739	2.72	2.741	2.741	2.718	2.723	2.721	2.721	2.745	2.746
G33	H3'	5.023	5.017	5.022	5.027	5.019	5.021	5.018	5.015	5.02	5.021
G33	H4'	4.431	4.427	---	---	---	---	---	---	---	---
G33	H5'	---	---	---	---	---	---	---	---	---	---
G33	H5''	---	---	---	---	---	---	---	---	---	---
G33	P	-4.402	-4.175	-4.157	-4.297	-4.194	-4.247	-4.26	-4.311	-4.254	-4.246
G33	H8	7.809	7.803	7.812	7.817	7.825	7.805	7.806	7.809	7.816	7.817
C34	H1'	5.794	5.743	5.743	5.745	5.768	5.742	5.736	5.736	5.741	5.734

C34	H2'	1.923	1.916	1.937	1.941	1.942	1.919	---	---	1.936	1.932
C34	H2''	2.367	2.349	2.366	2.371	2.377	2.35	2.345	2.341	2.366	2.361
C34	H3'	4.834	4.831	4.847	4.851	4.841	4.833	4.831	4.831	4.845	4.845
C34	H4'	4.172	---	---	---	---	---	---	---	---	---
C34	H5'	---	---	---	---	---	---	---	---	---	---
C34	H5''	---	---	---	---	---	---	---	---	---	---
C34	P	-4.231	-4.072	-4.017	-4.113	-4.044	-4.079	-4.076	-4.078	-4.101	-4.08
C34	H5	5.448	5.443	5.442	5.453	5.45	5.45	5.447	5.448	5.446	5.447
C34	H6	7.327	7.321	7.339	7.346	7.346	7.321	7.317	7.315	7.336	7.335
G35	H1'	6.188	6.173	6.195	6.198	6.189	6.172	6.167	6.167	6.196	6.194
G35	H2'	2.633	2.628	2.64	2.646	2.637	2.63	2.629	2.628	2.64	2.64
G35	H2''	2.404	2.389	2.382	2.386	2.38	2.39	2.389	2.389	2.383	2.385
G35	H3'	4.699	4.698	4.706	4.712	4.704	4.702	4.699	4.699	4.707	4.705
G35	H4'	4.195	---	---	---	---	---	---	---	---	---
G35	H5'	4.104	---	---	---	---	---	---	---	---	---
G35	H5''	---	---	---	---	---	---	---	---	---	---
G35	P	-4.095	-3.919	-3.85	-3.912	-3.942	-3.934	-3.927	-3.932	-3.92	-3.887
G35	H8	7.949	7.947	7.973	7.975	7.97	7.947	7.942	7.942	7.973	7.974

[a]none among the H5’/H5’’ pairs were assigned stereospecifically

[b]the H2’/H2’’ pairs for residues: 1, 2, 3, 4, 5, 10, 11, 12, 14, 15, 16, 17, 18, 19, 21, 23, 24, 25, 27, 31, 32, 33, 34 and 35 were assigned stereospecifically
