# Supplementary material for: The 8-17 DNAzyme can operate in a single active structure regardless of metal ion cofactor
Source: Nat Commun. 2024 May 17;15:4218. doi: 10.1038/s41467-024-48638-x (PMC11101458; doi:10.1038/s41467-024-48638-x)
Supplement: Supplementary file 5 — Supplementary Data 2 [file 41467_2024_48638_MOESM5_ESM.pdf]

Supplementary Data 2. Exchangeable $^{1}H$ chemical shifts measured for 8-17_short in sodium cacodylate buffer (pH 6.0) in the presence of different concentrations of divalent and monovalent ions.

| | | Sample conditions | | | | | | | | | |
|---|---|---|---|---|---|---|---|---|---|---|---|
| Metal ions present: | | 4 mM $Zn^{2+}$, 200 mM $Na^{+}$ | 400 mM $Na^{+}$ | 1.5 mM $Zn^{2+}$ | 7.5 mM $Mg^{2+}$ | 3.5 mM $Pb^{2+}$ | 400 mM $Na^{+}$, 1.5 mM $Zn^{2+}$ | 400 mM $Na^{+}$, 3.0 mM $Zn^{2+}$ | 400 mM $Na^{+}$, 4.5 mM $Zn^{2+}$ | 3.0 mM $Zn^{2+}$ | 4.5 mM $Zn^{2+}$ |
| DNA concentration: | | 2.2 mM | 1.5 mM | 1.5 mM | 1.5 mM | 1.5 mM | 1.5 mM | 1.5 mM | 1.5 mM | 1.5 mM | 1.5 mM |
| Temperature: | | 35 °C | 20 °C | 15 °C | 15 °C | 15 °C | 20 °C | 20 °C | 20 °C | 15 °C | 15 °C |
| Comment: | | conditions used for structure determination | DNAzyme folding with $Na^{+}$ only | DNAzyme folding with $Zn^{+}$ only | DNAzyme folding with $Na^{+}$ only | DNAzyme folding with $Zn^{+}$ only | titration of the $Na^{+}$-stabilized DNAzyme with $Zn^{2+}$ | | | titration of the $Zn^{2+}$-stabilized DNAzyme with additional equivalents of the ion | |
| Residue | Atom | | | | | | | | | | |
| G2 | HN | 13.107 | 13.099 | 13.125 | 13.126 | 13.134 | 13.1 | 13.096 | 13.095 | 13.127 | 13.129 |
| C3 | HN41 | 8.19 | 8.202 | 8.247 | 8.242 | 8.2 | 8.186 | 8.165 | 8.143 | 8.225 | 8.215 |
| C3 | HN42 | 6.458 | 6.423 | 6.451 | 6.438 | 6.491 | 6.429 | 6.438 | 6.448 | 6.47 | 6.476 |
| C4 | HN41 | 8.45 | 8.52 | 8.52 | 8.526 | 8.492 | 8.493 | 8.455 | 8.414 | 8.473 | 8.462 |
| C4 | HN42 | 6.922 | 6.901 | 6.938 | 6.944 | 6.945 | 6.899 | 6.904 | 6.907 | 6.95 | 6.959 |
| G5 | HN | 12.917 | 12.896 | 12.911 | 12.898 | 12.9 | 12.909 | 12.907 | 12.907 | 12.918 | 12.923 |
| G6 | HN | --- | --- | --- | --- | --- | --- | --- | --- | --- | --- |
| G7 | HN | 11.708 | 11.214 | 11.646 | 11.35 | 11.424 | 11.663 | 11.678 | 11.695 | 11.687 | 11.687 |
| G8 | HN | 12.797 | 12.605 | 12.777 | 12.734 | 12.765 | 12.76 | 12.787 | 12.797 | 12.801 | 12.808 |
| T9 | HN | 13.913 | 13.862 | 13.91 | 13.962 | 13.941 | 13.901 | 13.909 | 13.91 | 13.91 | 13.921 |
| C10 | HN41 | 8.5 | 8.497 | 8.505 | 8.5 | 8.495 | 8.5 | 8.5 | 8.495 | 8.502 | 8.499 |
| C10 | HN42 | 7.146 | 7.119 | 7.157 | 7.133 | 7.146 | 7.128 | 7.128 | 7.121 | 7.159 | 7.163 |
| G11 | HN | --- | --- | --- | --- | --- | --- | --- | --- | --- | --- |
| G14 | HN | 12.967 | 12.963 | 12.977 | 12.974 | 12.986 | 12.955 | 12.955 | 12.951 | 12.983 | 12.987 |
| C16 | HN41 | 8.1 | 8.135 | 8.106 | 8.081 | 8.14 | 8.102 | 8.098 | 8.096 | 8.107 | 8.107 |
| C16 | HN42 | 6.725 | 6.614 | 6.583 | 6.568 | 6.628 | 6.542 | 6.525 | 6.523 | 6.576 | 6.578 |
| T17 | HN | 10.743 | 11.134 | 10.801 | 11.07 | 10.914 | 10.84 | 10.756 | 10.723 | 10.753 | 10.752 |

| | | | | | | | | | | | |
|---|---|---|---|---|---|---|---|---|---|---|---|
| G18 | HN | 12.911 | 12.812 | 12.889 | --- | --- | 12.901 | 12.915 | 12.925 | 12.909 | 12.923 |
| C19 | HN41 | 7.736 | 7.808 | 7.784 | 8.013 | 7.862 | 7.742 | 7.716 | 7.699 | 7.764 | 7.755 |
| C19 | HN42 | 6.716 | 6.608 | 6.698 | 6.687 | 6.663 | 6.68 | 6.701 | --- | 6.715 | 6.718 |
| C20 | HN41 | 8.918 | 8.924 | 8.97 | 8.995 | 8.931 | 8.939 | 8.898 | 8.848 | 8.907 | 8.867 |
| C20 | HN42 | 7.075 | 6.952 | 7.06 | 7.044 | 7.067 | 6.971 | 6.985 | 6.985 | 7.058 | 7.017 |
| G22 | HN | 13.162 | 13.138 | 13.154 | 13.154 | --- | 13.13 | 13.128 | 13.129 | 13.161 | 13.152 |
| C23 | H41/H42[a] | 8.986 | 8.438 | 8.848 | --- | --- | --- | --- | --- | 8.775 | 8.462 |
| G24 | HN | 13.216 | 13.137 | 13.186 | 13.182 | 13.164 | 13.205 | 13.242 | 13.276 | 13.235 | 13.278 |
| G25 | HN | 12.532 | 12.587 | 12.547 | 12.639 | --- | 12.516 | 12.511 | 12.523 | 12.56 | 12.567 |
| C26 | HN41 | 8.055 | 8.125 | 8.093 | 8.184 | 8.175 | 8.061 | 8.044 | 8.038 | 8.077 | 8.078 |
| C26 | HN42 | 6.799 | 6.707 | 6.796 | 6.726 | 6.774 | 6.763 | 6.776 | 6.777 | 6.804 | 6.804 |
| T27 | HN | 10.808 | --- | --- | --- | --- | --- | --- | --- | --- | --- |
| C28 | HN41 | 8.687 | 8.741 | 8.689 | 8.699 | 8.698 | 8.689 | 8.683 | 8.687 | 8.698 | 8.707 |
| C28 | HN42 | 7.271 | 7.317 | 7.256 | 7.306 | 7.34 | 7.243 | 7.239 | 7.24 | 7.256 | 7.258 |
| G29 | HN | 10.973 | --- | --- | --- | --- | --- | --- | --- | --- | --- |
| C31 | HN41 | 8.488 | 8.479 | 8.5 | 8.467 | 8.47 | 8.494 | 8.491 | 8.488 | 8.5 | 8.505 |
| C31 | HN42 | 7.041 | 7.007 | 7.041 | 6.998 | 7.02 | 7.028 | 7.034 | 7.039 | 7.058 | 7.07 |
| G32 | HN | 13.235 | 13.188 | 13.2 | 13.208 | 13.24 | 13.204 | 13.221 | 13.241 | 13.23 | 13.24 |
| G33 | HN | 13.051 | 13.016 | 13.046 | 13.06 | 13.082 | 13.027 | 13.034 | 13.044 | 13.062 | 13.069 |
| C34 | HN41 | 8.459 | 8.455 | 8.502 | 8.502 | 8.462 | 8.451 | 8.437 | 8.427 | 8.494 | 8.494 |
| C34 | HN42 | 6.651 | 6.628 | 6.658 | 6.669 | 6.67 | 6.633 | 6.631 | 6.631 | 6.665 | 6.669 |

[a]these protons appeared as a single resonance
