# Supplementary material for: The 8-17 DNAzyme can operate in a single active structure regardless of metal ion cofactor
Source: Nat Commun. 2024 May 17;15:4218. doi: 10.1038/s41467-024-48638-x (PMC11101458; doi:10.1038/s41467-024-48638-x)
Supplement: Supplementary file 6 — Reporting Summary [file 41467_2024_48638_MOESM6_ESM.pdf]

nature portfolio

Corresponding author(s): Witold Andrałojć

Last updated by author(s): Apr 25, 2024

Reporting Summary

Nature Portfolio wishes to improve the reproducibility of the work that we publish. This form provides structure for consistency and transparency in reporting. For further information on Nature Portfolio policies, see our Editorial Policies and the Editorial Policy Checklist.

Statistics

For all statistical analyses, confirm that the following items are present in the figure legend, table legend, main text, or Methods section.

n/a	Confirmed	
☐	☒	The exact sample size (n) for each experimental group/condition, given as a discrete number and unit of measurement
☐	☒	A statement on whether measurements were taken from distinct samples or whether the same sample was measured repeatedly
☒	☐	The statistical test(s) used AND whether they are one- or two-sided<br>*Only common tests should be described solely by name; describe more complex techniques in the Methods section.*
☒	☐	A description of all covariates tested
☒	☐	A description of any assumptions or corrections, such as tests of normality and adjustment for multiple comparisons
☐	☒	A full description of the statistical parameters including central tendency (e.g. means) or other basic estimates (e.g. regression coefficient) AND variation (e.g. standard deviation) or associated estimates of uncertainty (e.g. confidence intervals)
☒	☐	For null hypothesis testing, the test statistic (e.g. F, t, r) with confidence intervals, effect sizes, degrees of freedom and P value noted<br>*Give P values as exact values whenever suitable.*
☒	☐	For Bayesian analysis, information on the choice of priors and Markov chain Monte Carlo settings
☒	☐	For hierarchical and complex designs, identification of the appropriate level for tests and full reporting of outcomes
☒	☐	Estimates of effect sizes (e.g. Cohen's d, Pearson's r), indicating how they were calculated

Our web collection on statistics for biologists contains articles on many of the points above.

Software and code

Policy information about availability of computer code

Data collection	Topspin 3.6, JASCO Spectra Manager 2.09.11
Data analysis	Topspin 3.6, NMRFAM-Sparky 1.470, AMBER 18, PyMol 1.6, ImageQuant TL

For manuscripts utilizing custom algorithms or software that are central to the research but not yet described in published literature, software must be made available to editors and reviewers. We strongly encourage code deposition in a community repository (e.g. GitHub). See the Nature Portfolio guidelines for submitting code & software for further information.

Data

Policy information about availability of data

All manuscripts must include a data availability statement. This statement should provide the following information, where applicable:

- Accession codes, unique identifiers, or web links for publicly available datasets
- A description of any restrictions on data availability
- For clinical datasets or third party data, please ensure that the statement adheres to our policy

Atomic coordinates and the list of experimental restraints for the reported NMR structure have been deposited with the Protein Data bank under accession code 8OR8 [https://doi.org/10.2210/pdb8OR8/pdb] while the chemical shifts have been deposited at the BMRB under the numbers 34805 [doi:10.13018/BMR34805] and 52355 [doi:10.13018/BMR52355]. The CD spectra and cleavage assays (gel scans) generated in this study are provided in the Source Data file. The raw NMR data used in this study are available in the Zenodo database under DOI: 10.5281/zenodo.11047570. This study also makes use of the previously published crystallographic structure: 5XM8 [https://doi.org/10.2210/pdb5XM8/pdb] for structural comparison.

Human research participants

Policy information about studies involving human research participants and Sex and Gender in Research.

Reporting on sex and gender	Not applicable
Population characteristics	Not applicable
Recruitment	Not applicable
Ethics oversight	Not applicable

Note that full information on the approval of the study protocol must also be provided in the manuscript.

Field-specific reporting

Please select the one below that is the best fit for your research. If you are not sure, read the appropriate sections before making your selection.

☒ Life sciences ☐ Behavioural & social sciences ☐ Ecological, evolutionary & environmental sciences

For a reference copy of the document with all sections, see nature.com/documents/nr-reporting-summary-flat.pdf

Life sciences study design

All studies must disclose on these points even when the disclosure is negative.

Sample size	No statistical methods were used to determine the sample sizes. When applicable the sample sizes were determined based on similar previously published studies. Namely, for the DNAzyme kinetics studies monitored by gel elecreophoresis it is a standard practice to perform 2-3 repetitions of the experiment to capture the errors arising from the limited precision of the operator during the experimental procedure (extracting sample aliquots at precisely determined points in time). As such, 3 repetitions were made for each such experiment reported in Supplementary Figure 2 (6 experimental condition, 3 repetitions in each conditions). After noticing the excellent reproducibility of these experiments in our hands (see Source data for Supplementary Figure 2) for some of the other sbsequently tested conditions only one replicate was performed (see Replication below).
Data exclusions	No data were excluded form the analysis.
Replication	For NMR and CD samples of the 8-17 DNAzyme over ten different batches of DNA were used, yielding virtually indistinguishible 1D spectra, confirming reproducibility of sample preparation and experimental conditions. For the first sets of kinetic assays, presented in Figure S2, each experiment was done in triplicate with excellent reproducibility. For the larger set of kinetic assays shown in Figure S3 the set of experimnets (for '8-17_short' in presence of Mg2+) was also done in triplicate, hovever after noticing excellent reproducibility between the replicates in these experiments for the other 5 series each condition was tested only once.
Randomization	This study did not allocate experimental groups, thus randomization is not required in the reported experiments.
Blinding	No experiments that require blinding were conducted within this study (no human, animal or behavioral experiments were performed in this study).

Reporting for specific materials, systems and methods

We require information from authors about some types of materials, experimental systems and methods used in many studies. Here, indicate whether each material, system or method listed is relevant to your study. If you are not sure if a list item applies to your research, read the appropriate section before selecting a response.

Materials & experimental systems

n/a	Involved in the study
☒	☐ Antibodies
☒	☐ Eukaryotic cell lines
☒	☐ Palaeontology and archaeology
☒	☐ Animals and other organisms
☒	☐ Clinical data
☒	☐ Dual use research of concern

Methods

n/a	Involved in the study
☒	☐ ChIP-seq
☒	☐ Flow cytometry
☒	☐ MRI-based neuroimaging
